# Supplementary material for: Unveiling the Dynamics behind Glioblastoma Multiforme Single-Cell Data Heterogeneity
Source: Int J Mol Sci. 2024 Apr 30;25(9):4894. doi: 10.3390/ijms25094894 (PMC11084314; doi:10.3390/ijms25094894)
Supplement: Supplementary file 1 [file ijms-25-04894-s001.zip › Supplementary_Materials.pdf]

Supplementary Materials for *Unveiling the dynamics behind Glioblastoma Multiforme single-cell data heterogeneity*

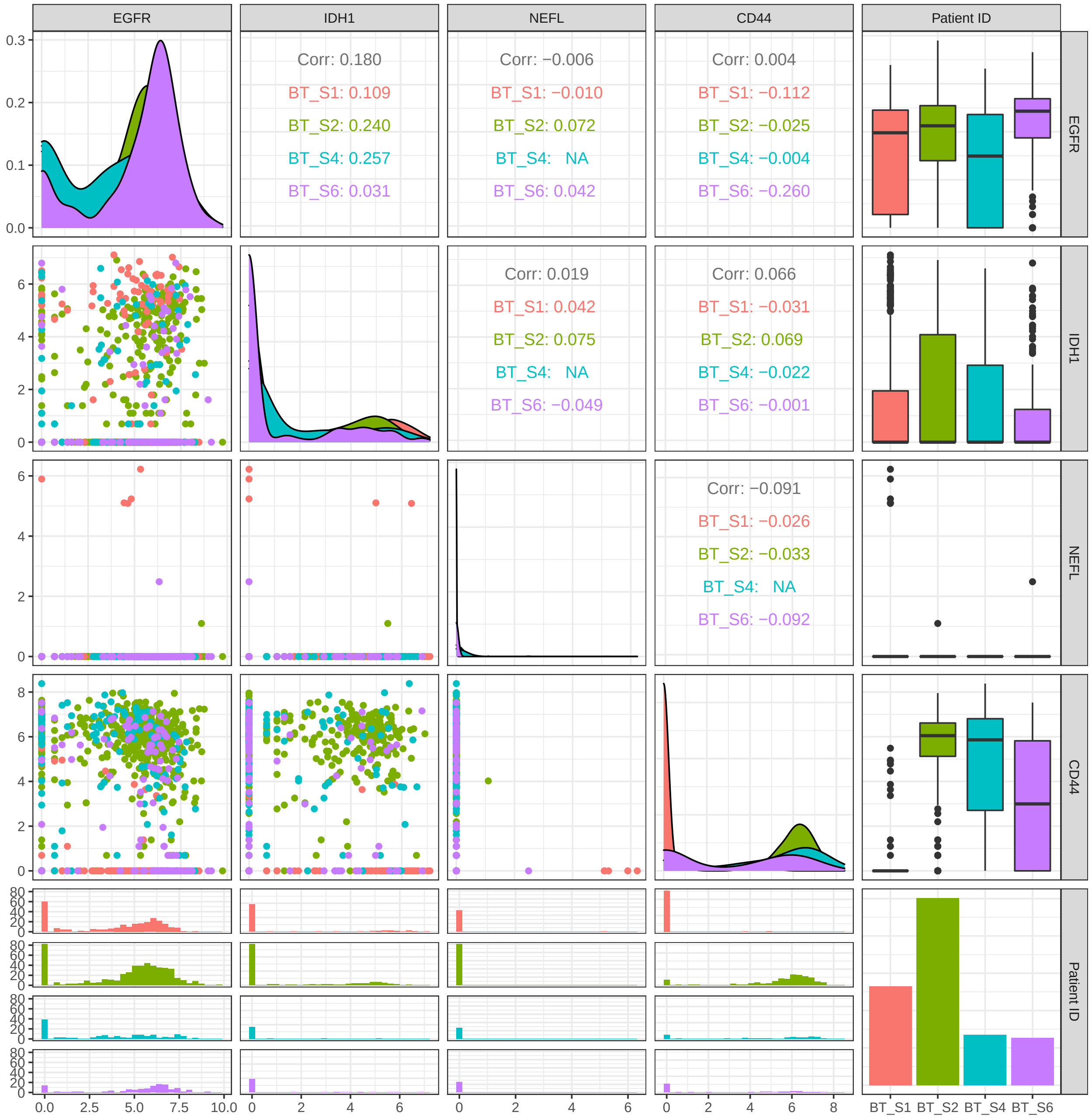

Figure S1. The figure presents a multifaceted exploration of the relationship between four selected genes—EGFR, IDH1, NEFL, and CD44—for the present scRNA-seq dataset. Each off-diagonal scatter plot showcases the pairwise relationship between two different genes, enabling an assessment of gene-gene relations. These scatter plots are color-coded based on 'Patient ID', providing additional information concerning inter-patient variability. The diagonal histograms present the distributions for each gene, highlighting their individual expression profiles. Above diagonal histograms, the Pearson correlation coefficients are displayed, offering a quantified measure of the linear relationship between each pair of genes. This comprehensive view allows for a multidimensional understanding of gene relations and their distributions across patients. The arrangement of markers in the scatter plots underscores a notable consistency in the spatial localization of these gene expression markers, irrespective of patient-to-patient variability. In the far-right column, boxplots for each gene across different patients provide an overview of the gene expression variability and central tendency within the patient cohort. The last row of this column features a bar chart that aggregates these data.

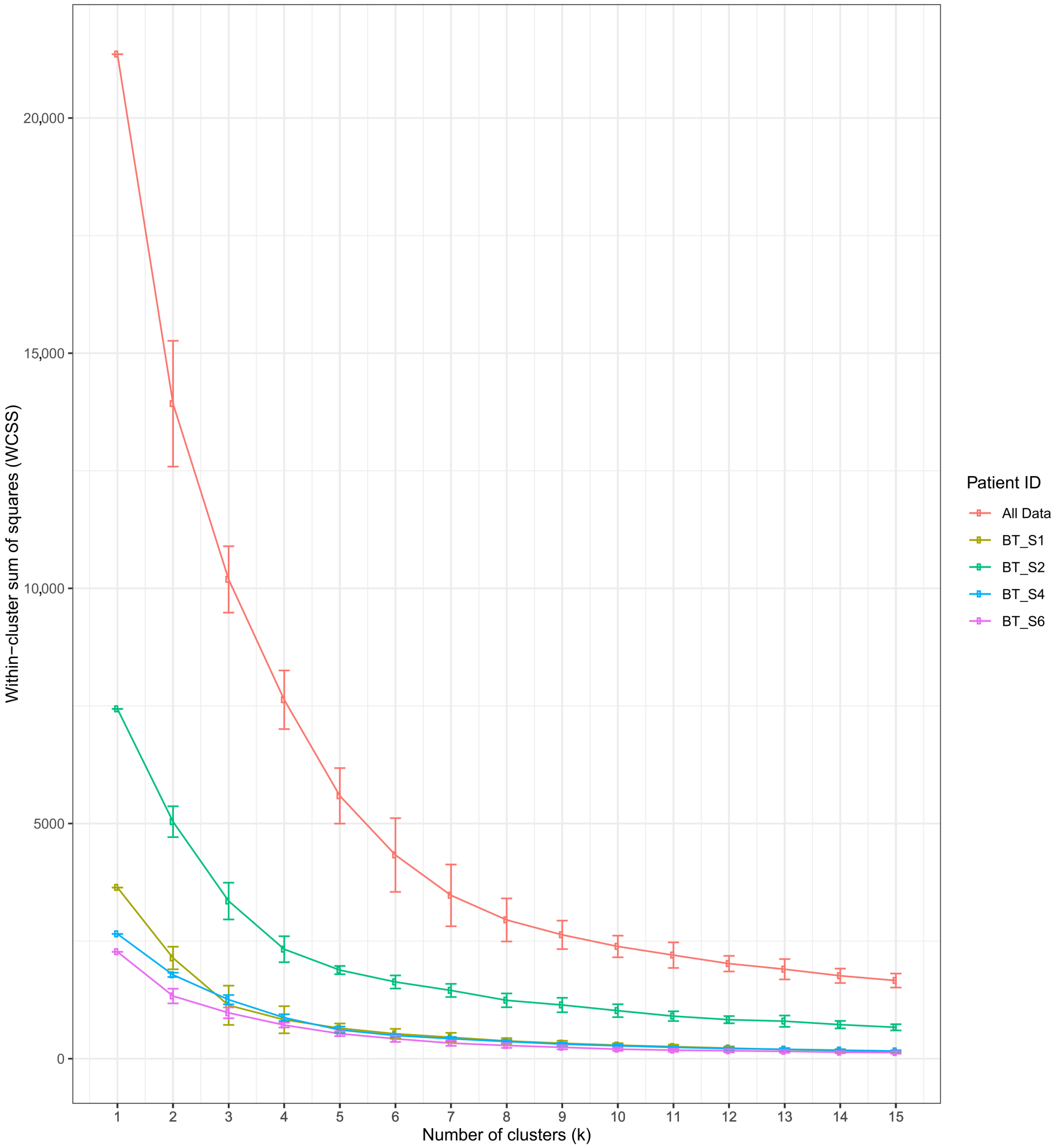

Figure S2. The figure presents an assessment of the Within-Cluster Sum of Squares (WCSS) to determine the optimal number of clusters (k) for the gene expression data, specifically focusing on four key genes—EGFR, IDH1, NEFL, and CD44. It encapsulates results derived from a repeated (100 runs) K-means clustering algorithm, both for individual patients and the aggregated dataset. Each data point represents the mean WCSS value computed across the 100 runs for each k value ranging from 1 to 15. Lines connect these mean points, providing a visual trajectory of WCSS as a function of the number of clusters. The error bars, extending from each mean point, represent the standard deviation of the WCSS values, thereby offering a glimpse into the algorithmic stability across different runs. This approach leverages both a global analysis (aggregated data) and local examinations (patient-specific data) to furnish a robust statistical landscape of the clustering behavior. The plot is color-coded based on 'Patient ID'.

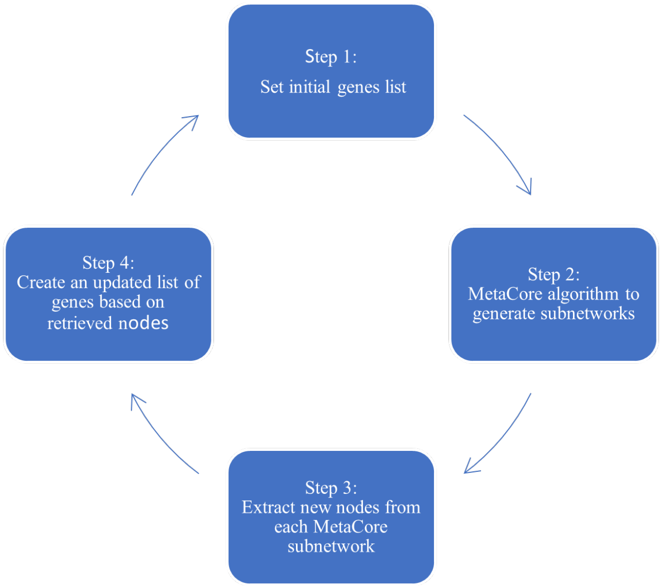

Figure S3. GRN building procedure: (i) Select a group of initial (marker) genes. (ii) Input the list into the MetaCore (accessed on 16 abril 2022) GRN generator. (iii) Retrieve additional vertices. (iv) Create a new list with the initial vertices and those added by the MetaCore algorithm. (v) Use the updated list as the initial group of genes. This cycle can be repeated until the network is interconnected or until no new vertices are added. The process was performed twice in this case, but the number of iterations can be adjusted according to specific requirements.

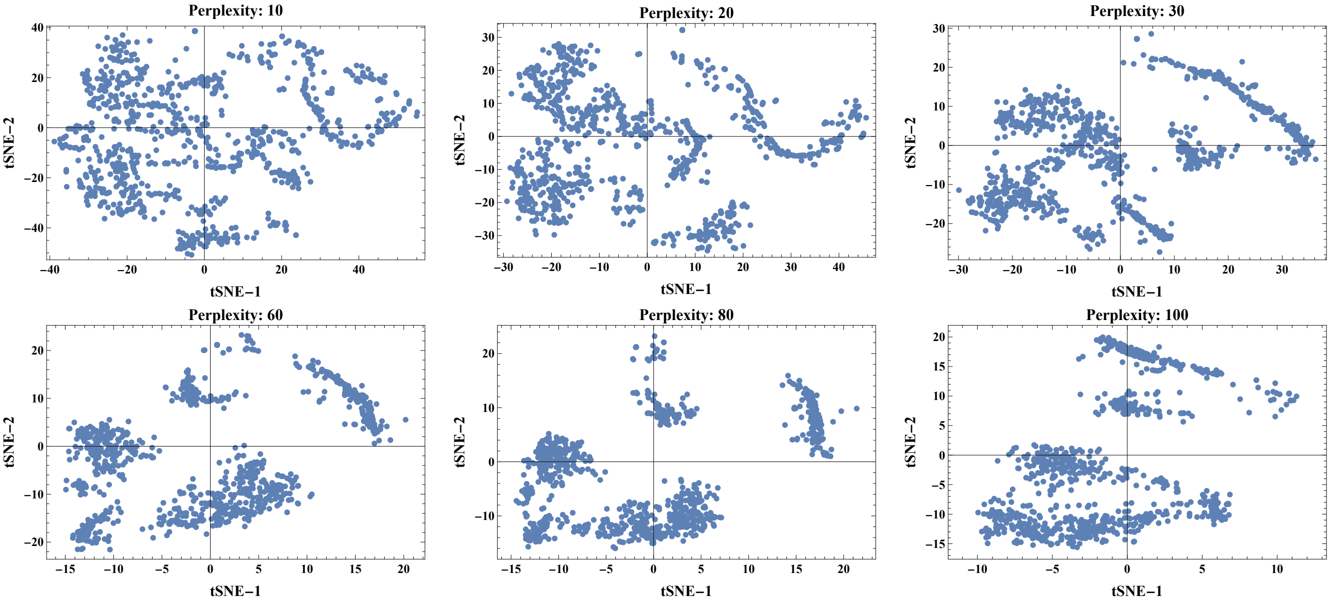

Figure S4. tSNE-based dimensional reduction visualizations with varying perplexity values (10, 20, 30, 60, 80, and 100). These plots demonstrate the effect of different perplexity values on the clustering and structure of the data, helping to identify the most suitable parameter for further analysis. The axes represent the two reduced dimensions obtained from tSNE.

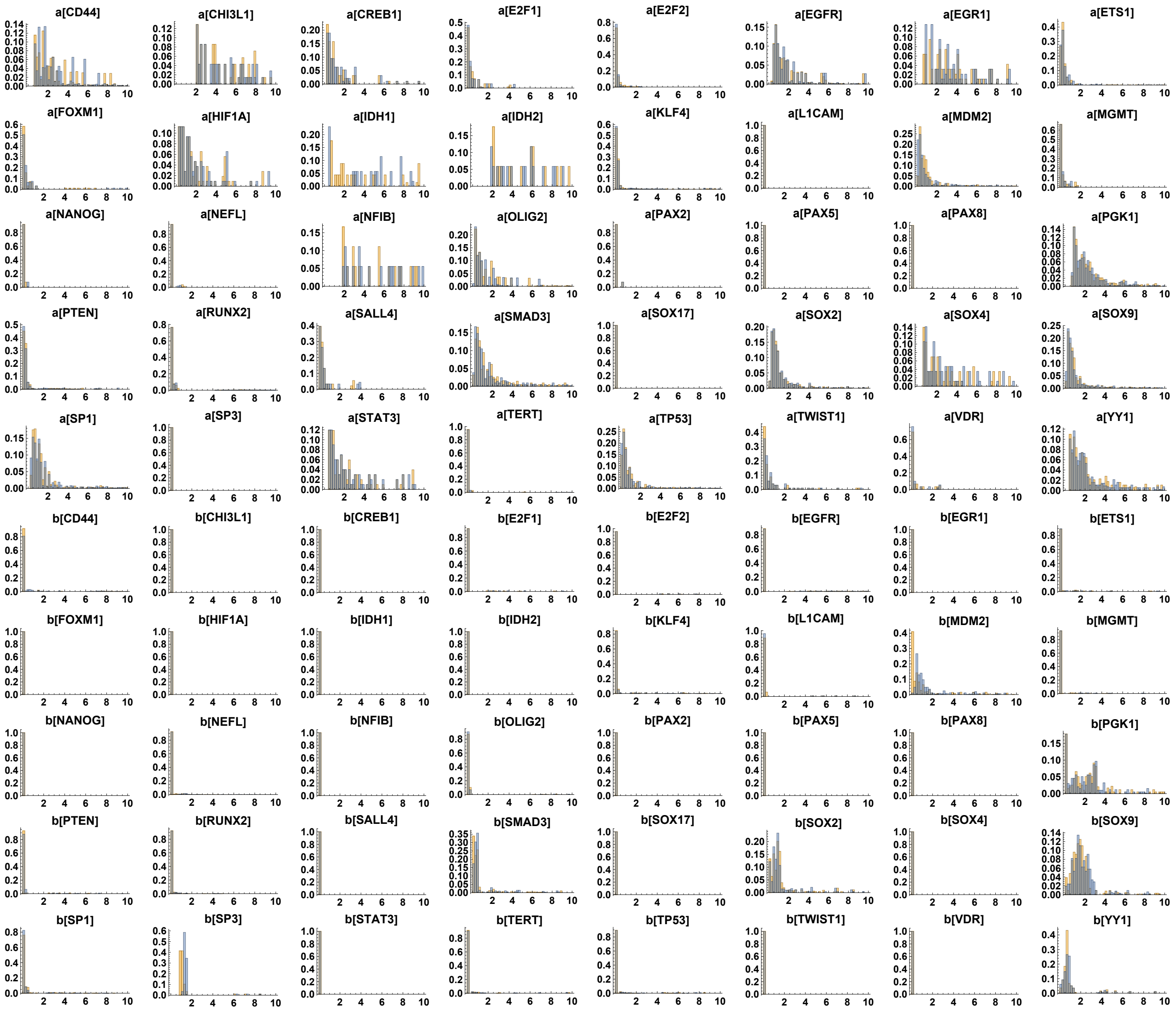

Figure S5. Distributions of the initial parameter estimation after clustering using k-means and NbC methods. The a[] and b[] notation refers to each gene's activation and inhibition parameters. The orange values represent k-means and the blue values represent NbC. These parameters multiply all interactions in each gene equation, with the same values applied across all considered clusters. The horizontal axis shows the parameter values, while the vertical axis represents normalized counts or frequencies to indicate probabilities.

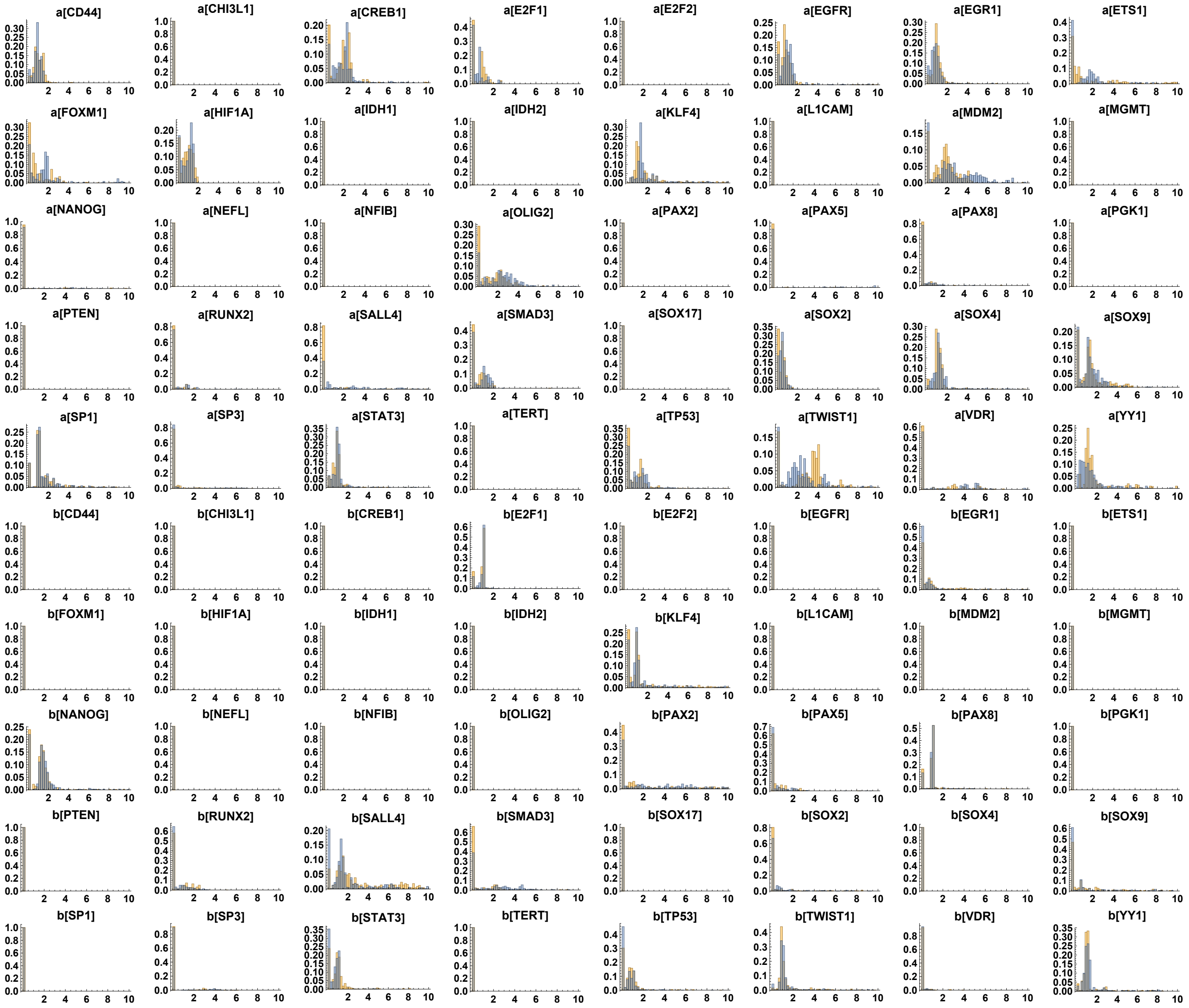

Figure S6. Distributions of the second parameter estimation after clustering using k-means and NbC methods. The a[] and b[] notation refers to each gene's activation and inhibition parameters. The orange values represent k-means and the blue values represent NbC. These second parameters multiply all interactions in all gene equations regulated by these edges. It multiplies the activation or inhibition parameters previously computed to balance the dynamics better. The horizontal axis shows the parameter values, while the vertical axis represents normalized counts or frequencies to indicate probabilities.

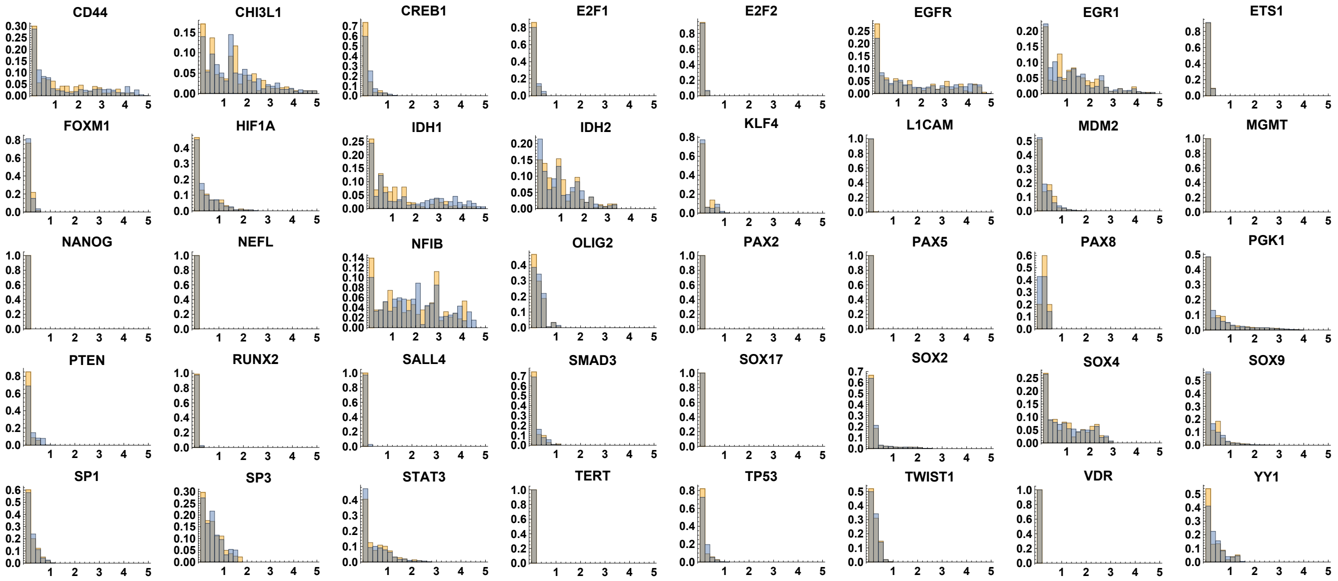

Figure S7. Superimposed residual distributions for the first parameters estimation after clustering using k-means and NbC methods. The orange values represent k-means and the blue values represent NbC. For each estimation, the number of residuals for each gene was equal to the number of clusters (due to considering all clustering simultaneously). The horizontal axis shows the residual values, while the vertical axis represents normalized counts or frequencies to indicate probabilities.

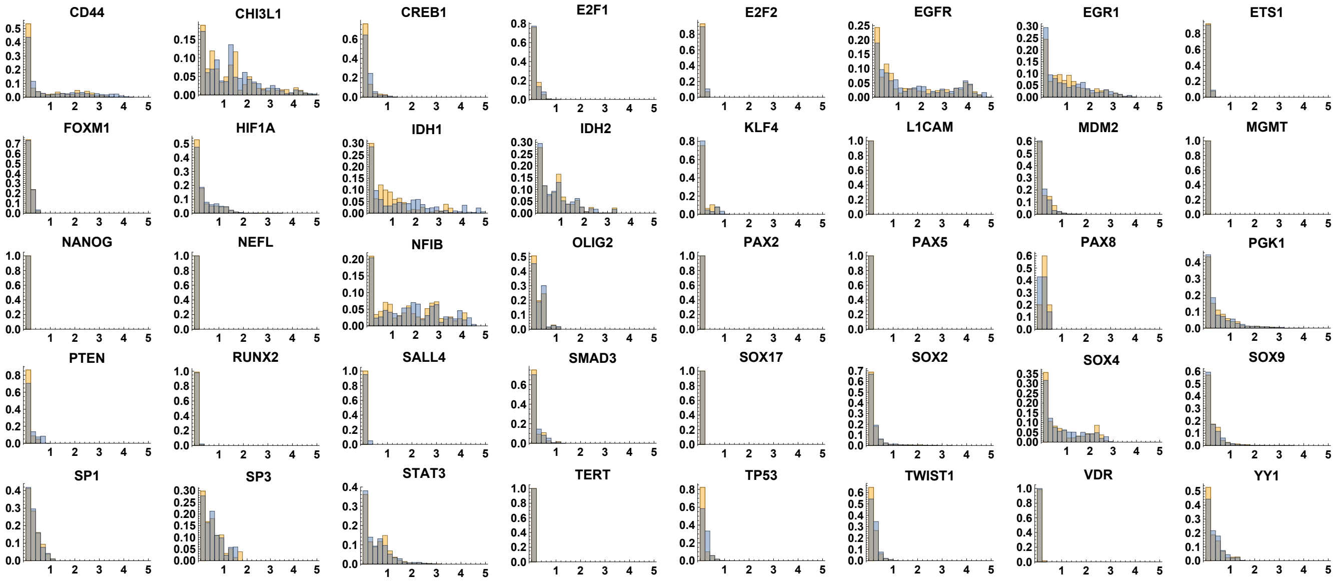

Figure S8. Superimposed residual distributions for the second parameters estimation after clustering using k-means and NbC methods. The orange values represent k-means and the blue values represent NbC. For each estimation, we had the number of residuals for each gene equal to the number of clusters (due to considering all clustering simultaneously). The horizontal axis shows the residual values, while the vertical axis represents normalized counts or frequencies to indicate probabilities.

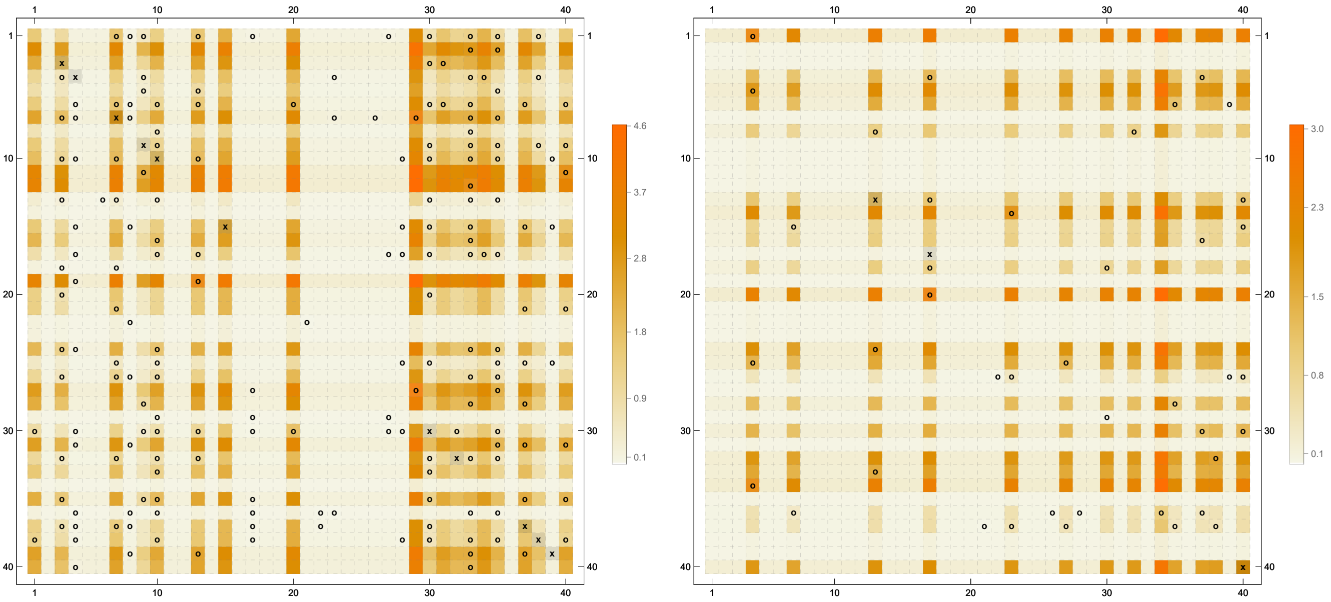

(**a**)

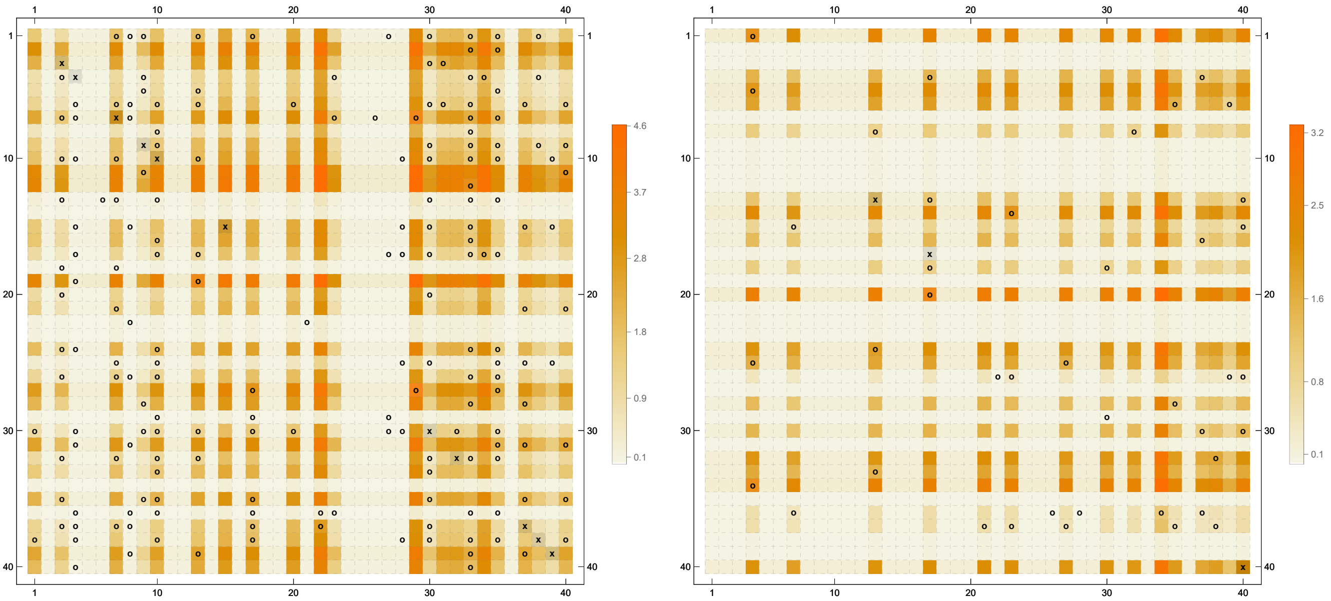

(**b**)

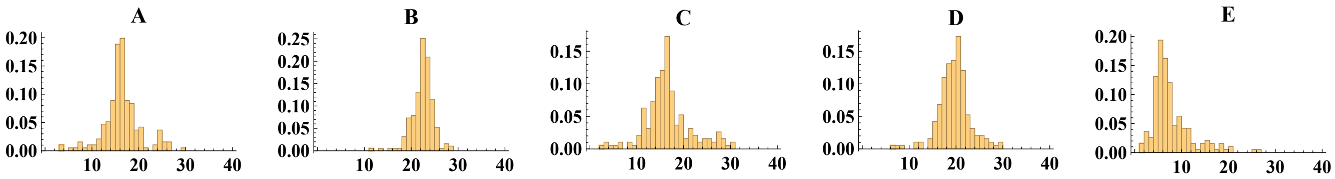

(**c**)

Figure S9. *Cont.*

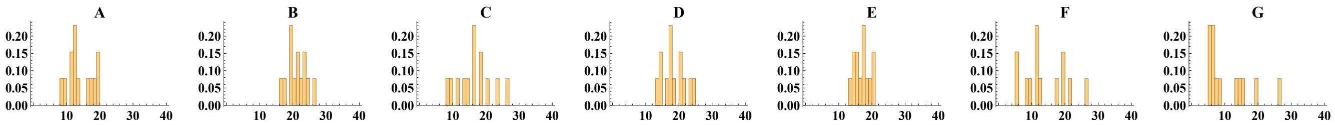

(**d**)

Figure S9. Figure (**a**,**b**) show the heatmap with the color gradient for the logarithm of the parameter values considering k-means and NbC, respectively. The 'o' represents the regulations and the 'x' is the self-regulation edges in the network. Left: activation parameters. Right: inhibition parameters. The matrix indices in (**a**,**b**) represent each variable/vertex of the model. Figure (**c**,**d**) represent the distribution of the number of genes that match each cluster, considering all tested combinations of the multiplicative factors for *a*, *sa*, *b*, and *sb*. The k-means clusters were labeled from A to E, and the NbC clusters from A to G. The horizontal axis represents the count of compatibility cases, and the vertical axis represents normalized counts or frequencies to indicate probabilities.

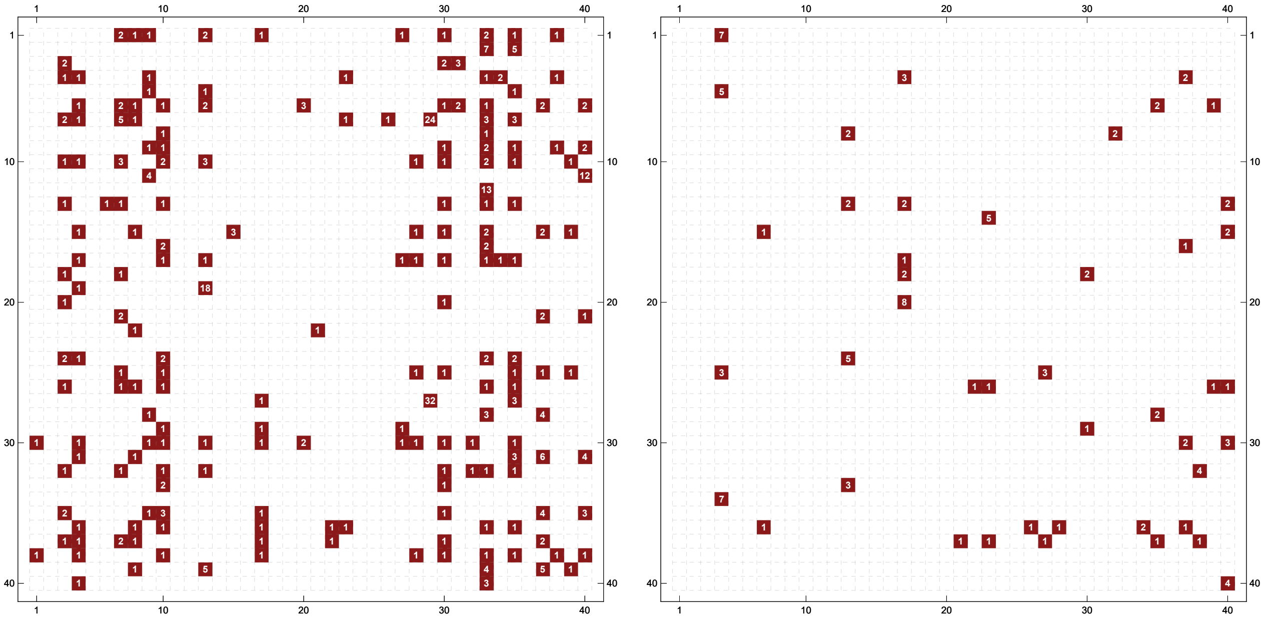

(**a**)

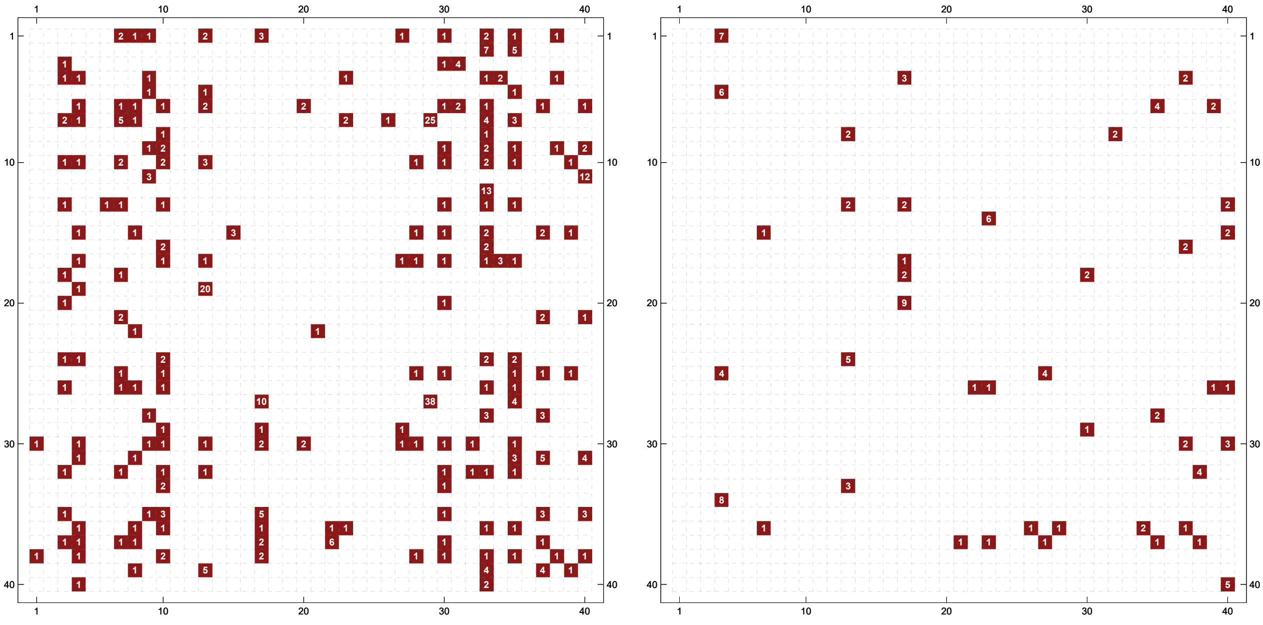

(**b**)

Figure S10. Figure (**a**,**b**) show the ceil() of the values of all parameters for the k-means clustering and NbC clustering, respectively. The left images correspond to activation parameters and the right images to inhibition parameters. The ceil() was used to help the visualization of the values. The matrix indices in (**a**,**b**) represent each variable/vertex of the model.

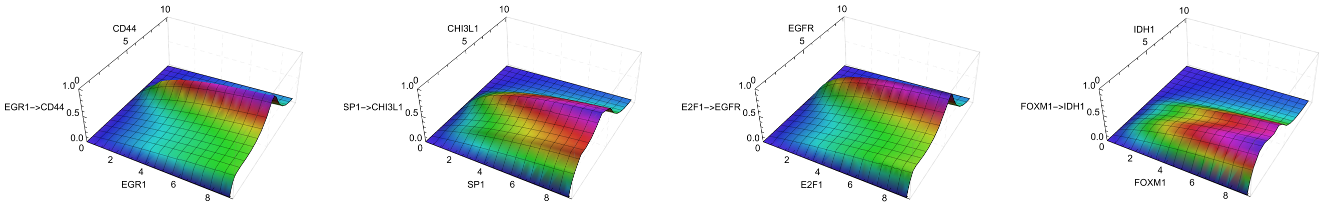

(**a**)

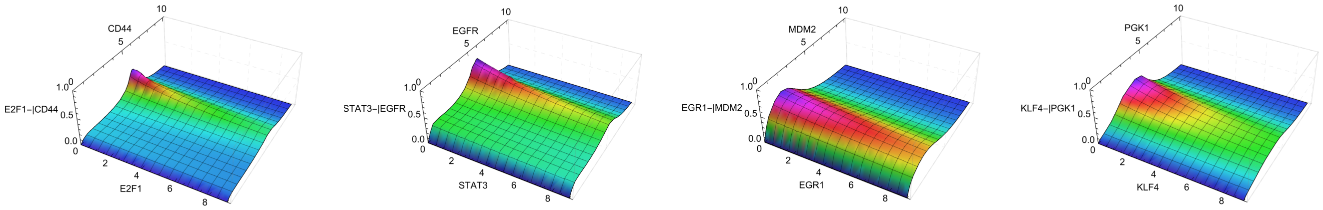

(**b**)

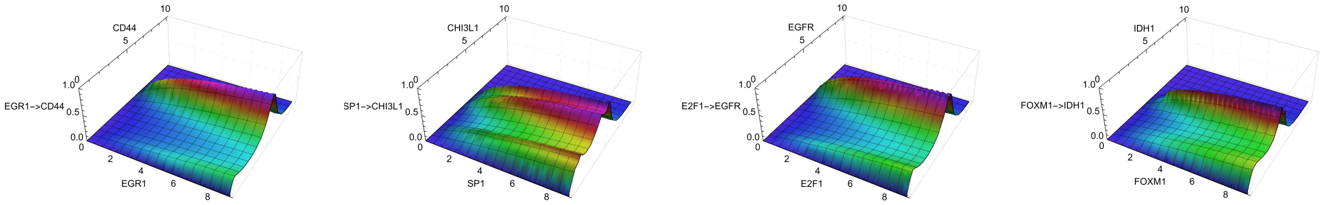

(**c**)

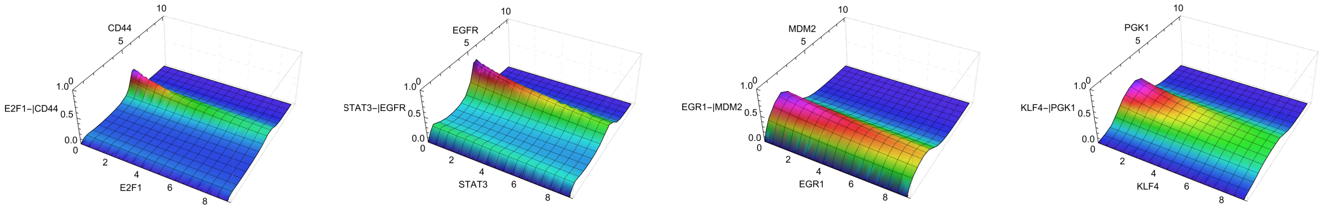

(**d**)

Figure S11. New regulation function V with $n = 1$ and $h_2(x)$ for different combinations of genes and/or transcription factors. The horizontal axis represents the transcription factor and gene quantification using the normalized amount of single-cell RNA sequencing of experimental data. The vertical axis represents the quantification of the interaction regulations. (**a**) Activation values using the five k-means clusters and $f_a = 0.1$. (**b**) Inhibitory interactions using the five k-means clusters and $f_b = 1.3$. (**c**) Activation values using the seven Neighborhood Contraction clusters and $f_a = 0.1$. (**d**) Inhibitory interactions using the seven Neighborhood Contraction clusters and $f_b = 1.1$.

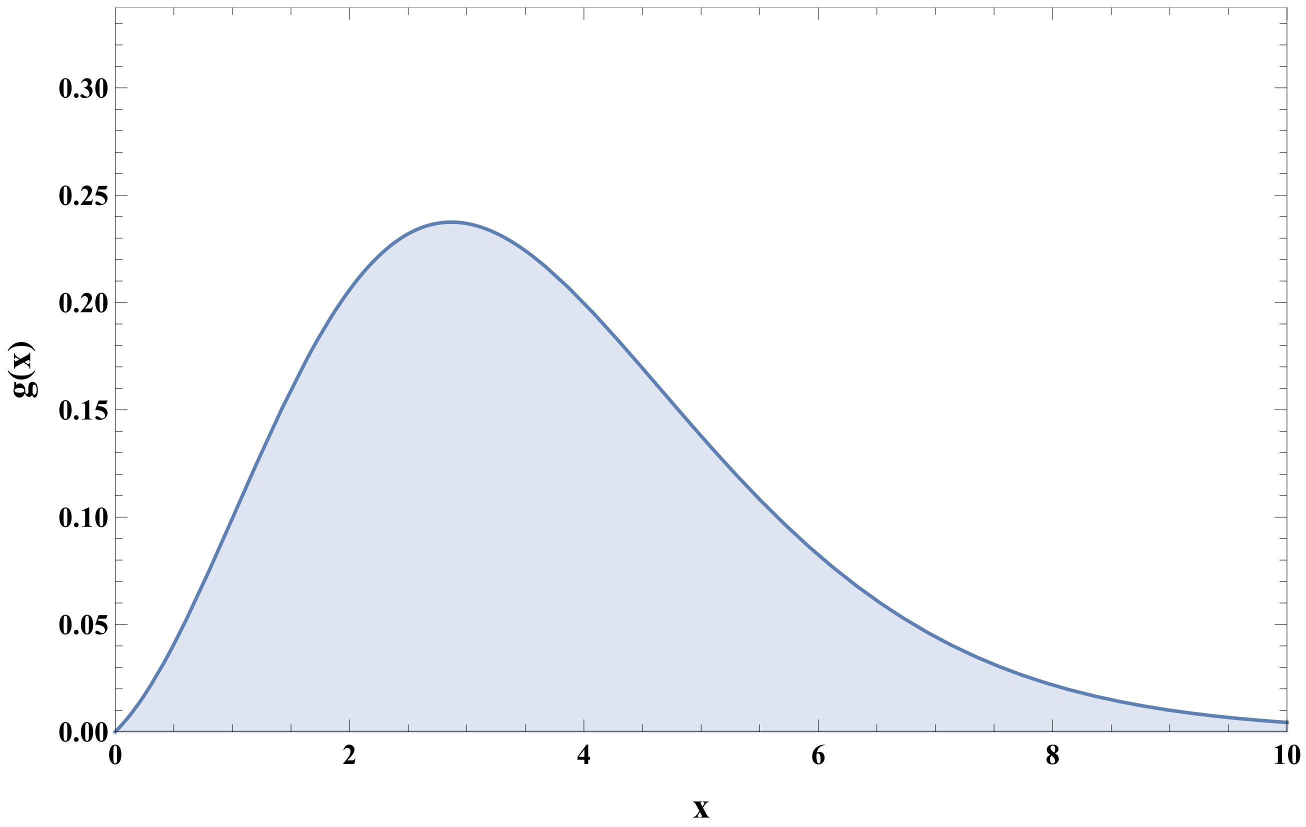

Figure S12. Function used for the multiplicative noise after manual verification of compatibility between the experimental data and those obtained by the simulation The horizontal axis represents the expression level quantification and the vertical axis represents the noise amplitude before multiplication by the amplitude that was defined in the parameter estimation.

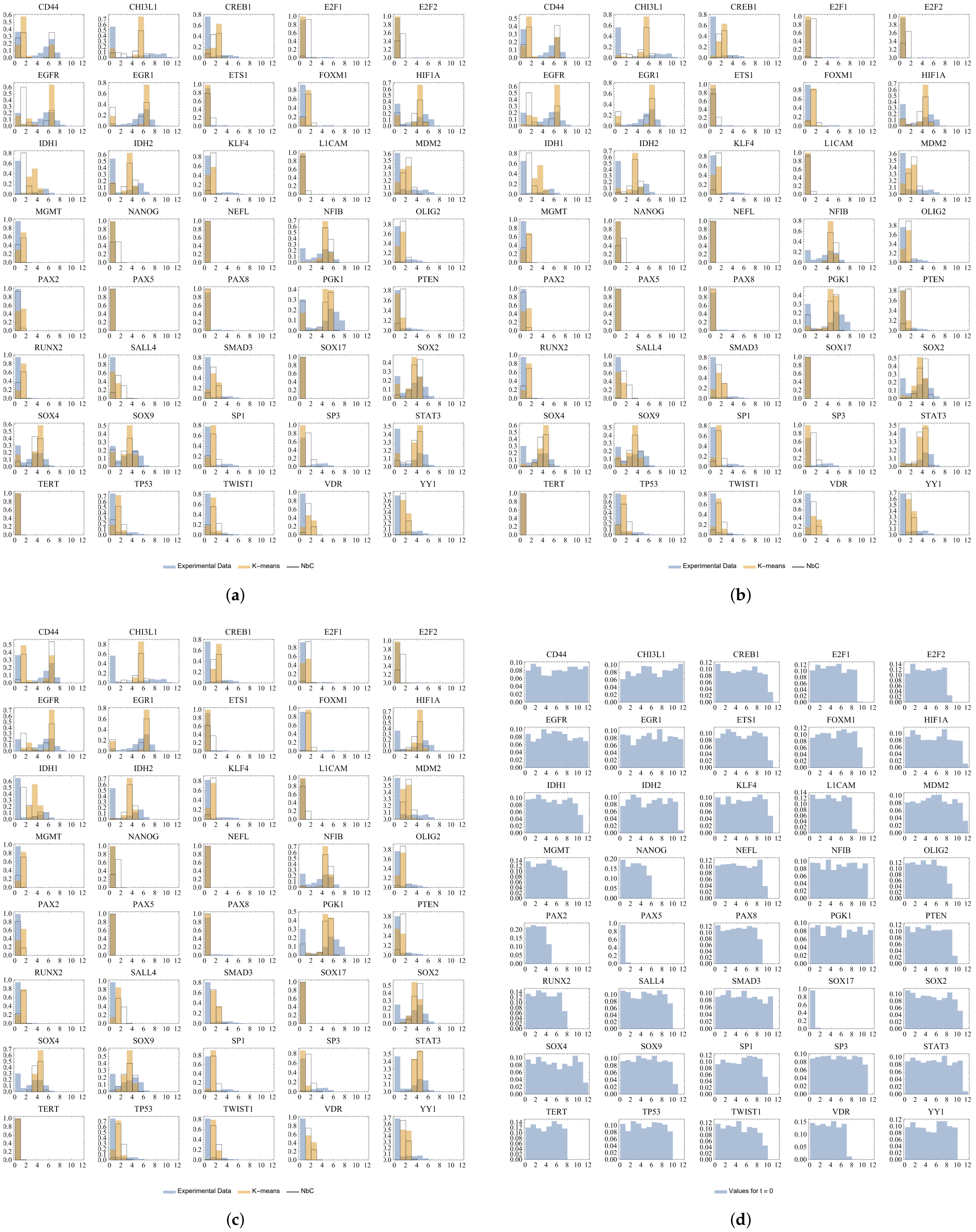

Figure S13. (**a**–**c**): Superposition of experimental (blue) and simulated (orange: K-means; black line: NbC) data for each gene of the GRN; (**a**) t = 50 (500 time steps); (**b**) t = 25 (250 time steps); (**c**) t = 5 (50 time steps); (**d**) distribution of simulated initial conditions. The expression values are on the x-axis, and the relative frequency in the y-axis. The interval goes from 0 to 12 in unity steps. The different times were used to analyze the probability distribution evolution over time. The horizontal axis shows the expression values, while the vertical axis represents normalized counts or frequencies to indicate probabilities.

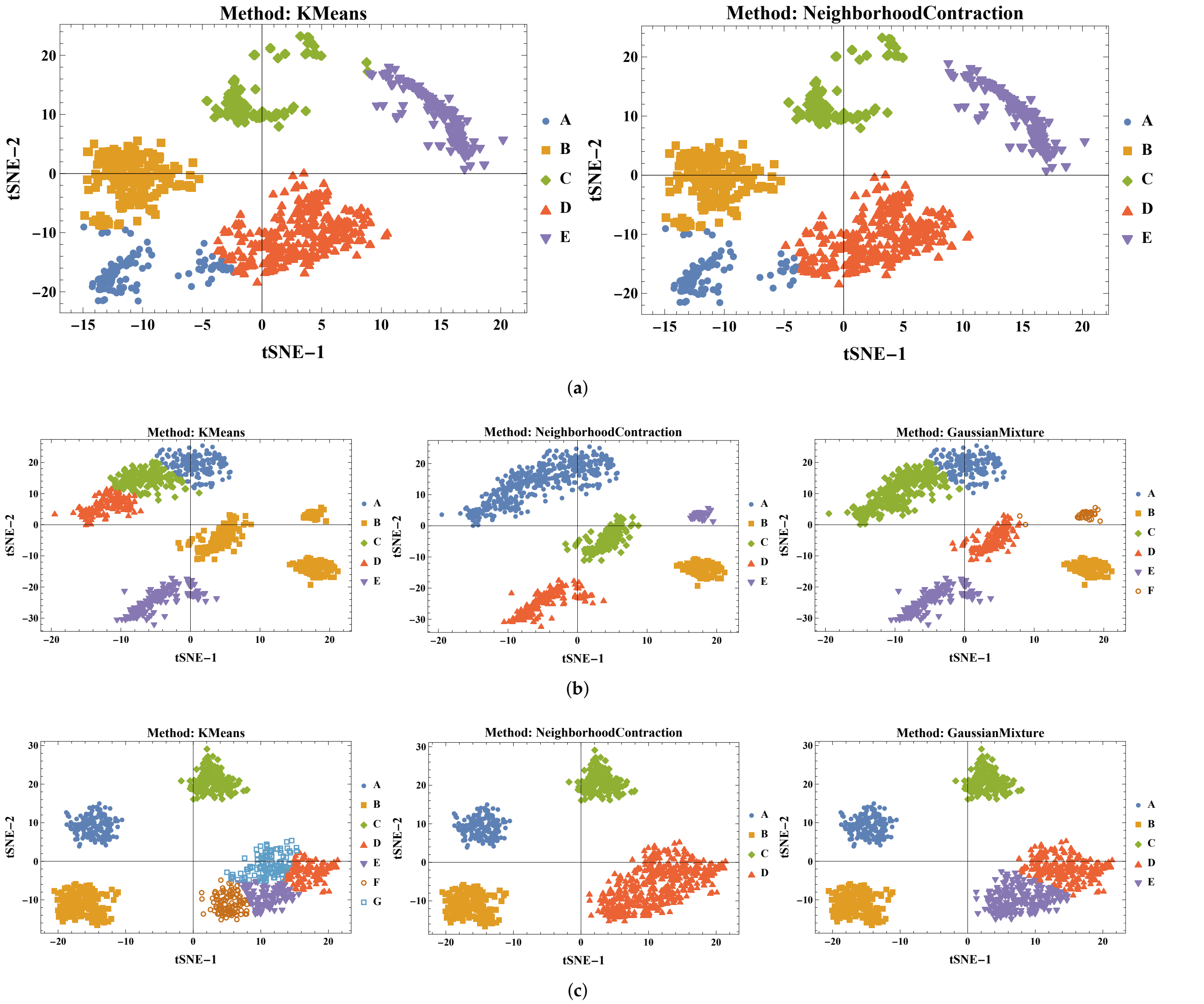

Figure S14. Clustering results using different methods. The axes represent the two reduced dimensions obtained for each tSNE. (**a**) Experimental data; (**b**) simulated data after k-means clustering; (**c**) simulated data after Nbc clustering. The letters represent the identified clusters. These clusters were defined in this two-dimensional space and were used solely to assess the clustering quality. The actual clustering was performed in the four-marker dimensional space. All methods used the built-in Mathematica functions with PerformanceGoal set to "Quality", CriterionFunction set to "StandardDeviation", and DistanceFunction set to EuclideanDistance.

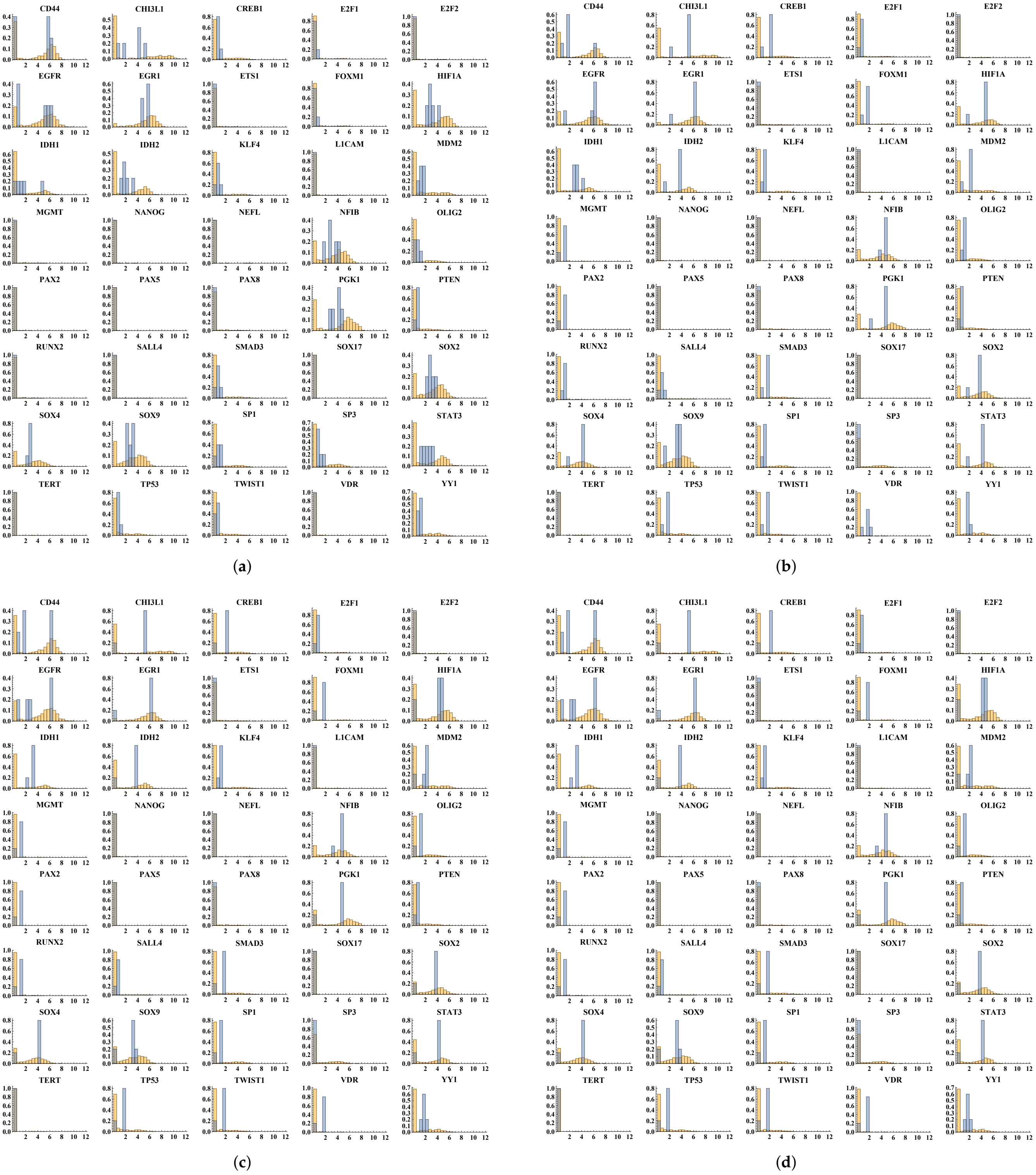

Figure S15. Cluster centroids superimposed on experimental data distributions for each gene. (**a**) Centroids found using k-means with experimental data; (**b**) centroids found using k-means with simulated data after parameter estimation using k-means clusters; (**c**) centroids found using NbC; (**d**) centroids found using Gaussian Mixture. The horizontal axis shows the expression values, while the vertical axis represents normalized counts or frequencies to indicate probabilities.

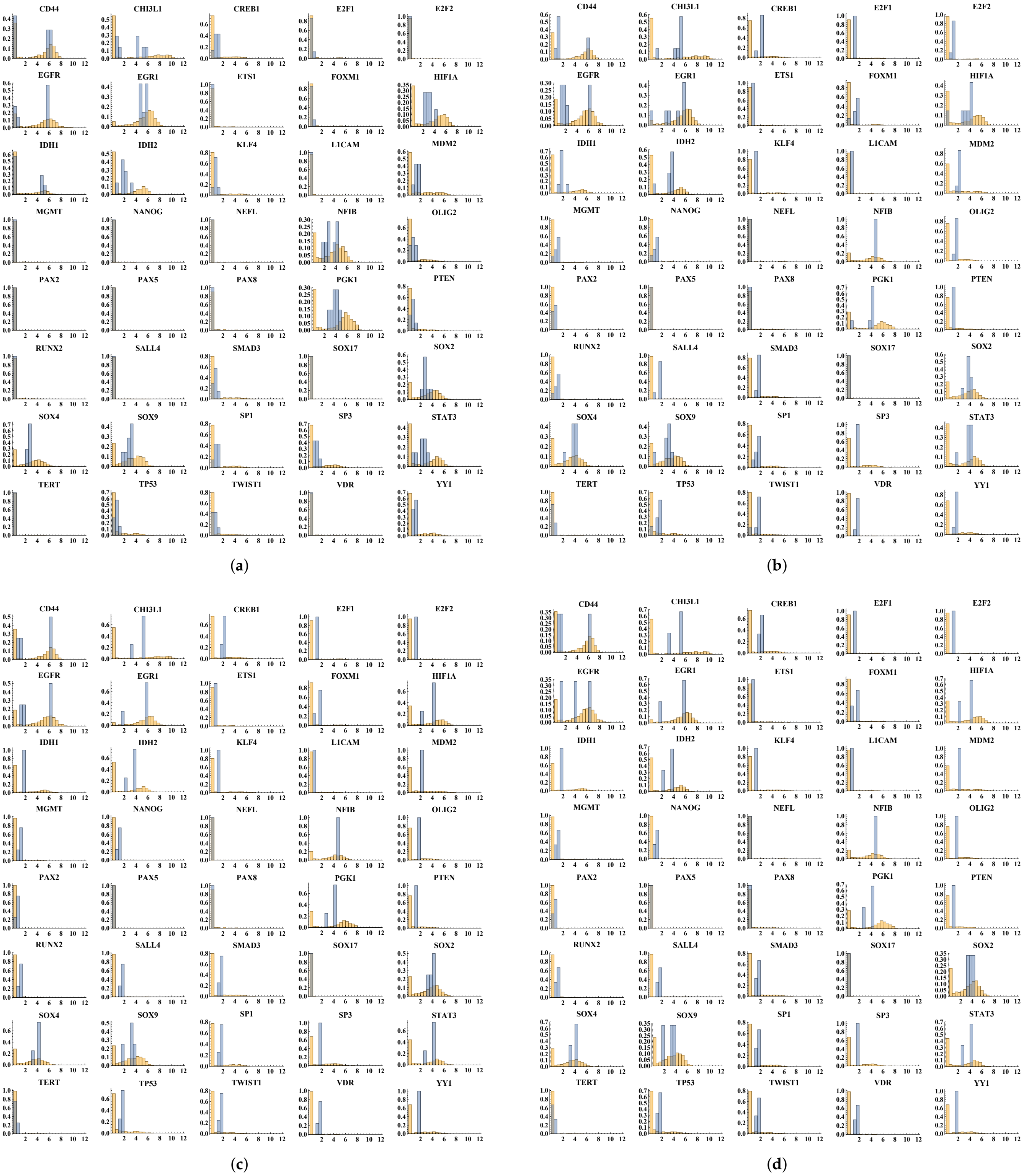

Figure S16. Cluster centroids superimposed on experimental data distributions for each gene. (**a**) Centroids found using NbC with experimental data; (**b**) centroids found using k-means with simulated data after parameter estimation using NbC clusters; (**c**) centroids found using NbC; (**d**) centroids found using Gaussian Mixture. The horizontal axis shows the expression values, while the vertical axis represents normalized counts or frequencies to indicate probabilities.

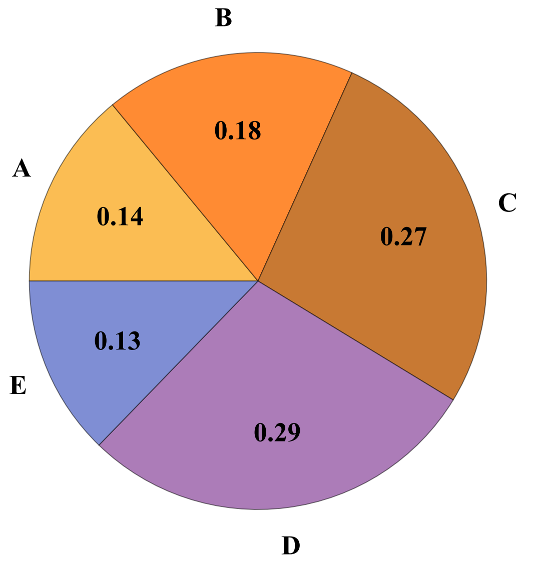

(**a**)

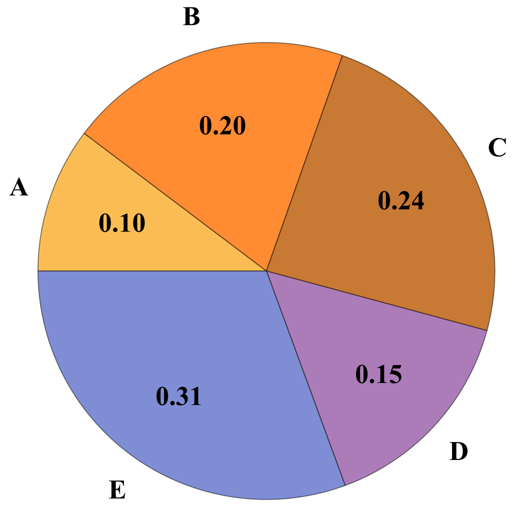

(**b**)

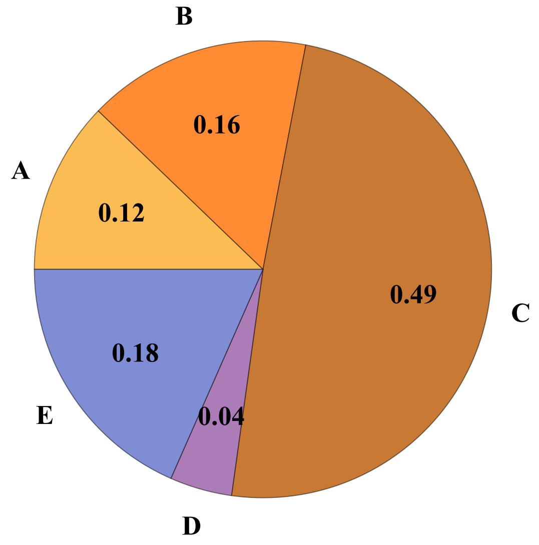

(**c**)

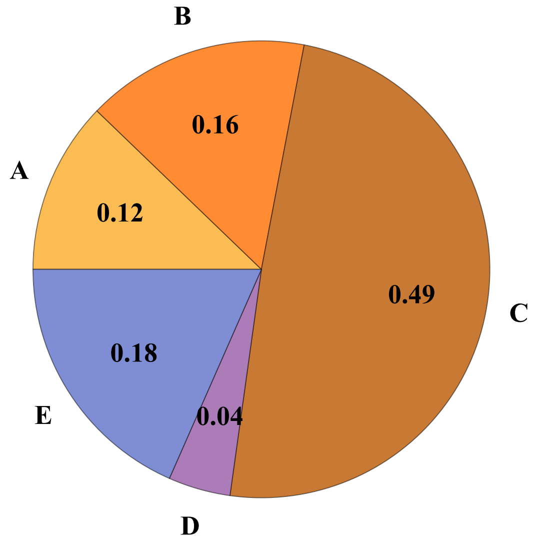

(**d**)

Figure S17. Pie charts representing the number of elements within each cluster, represented by the respective letters. (**a**) Experimental data clusters using k-means; (**b**–**d**) clusters of simulated data after parameter estimation using k-means clusters. (**b**) k-means; (**c**) NbC; (**d**) Gaussian Mixture.

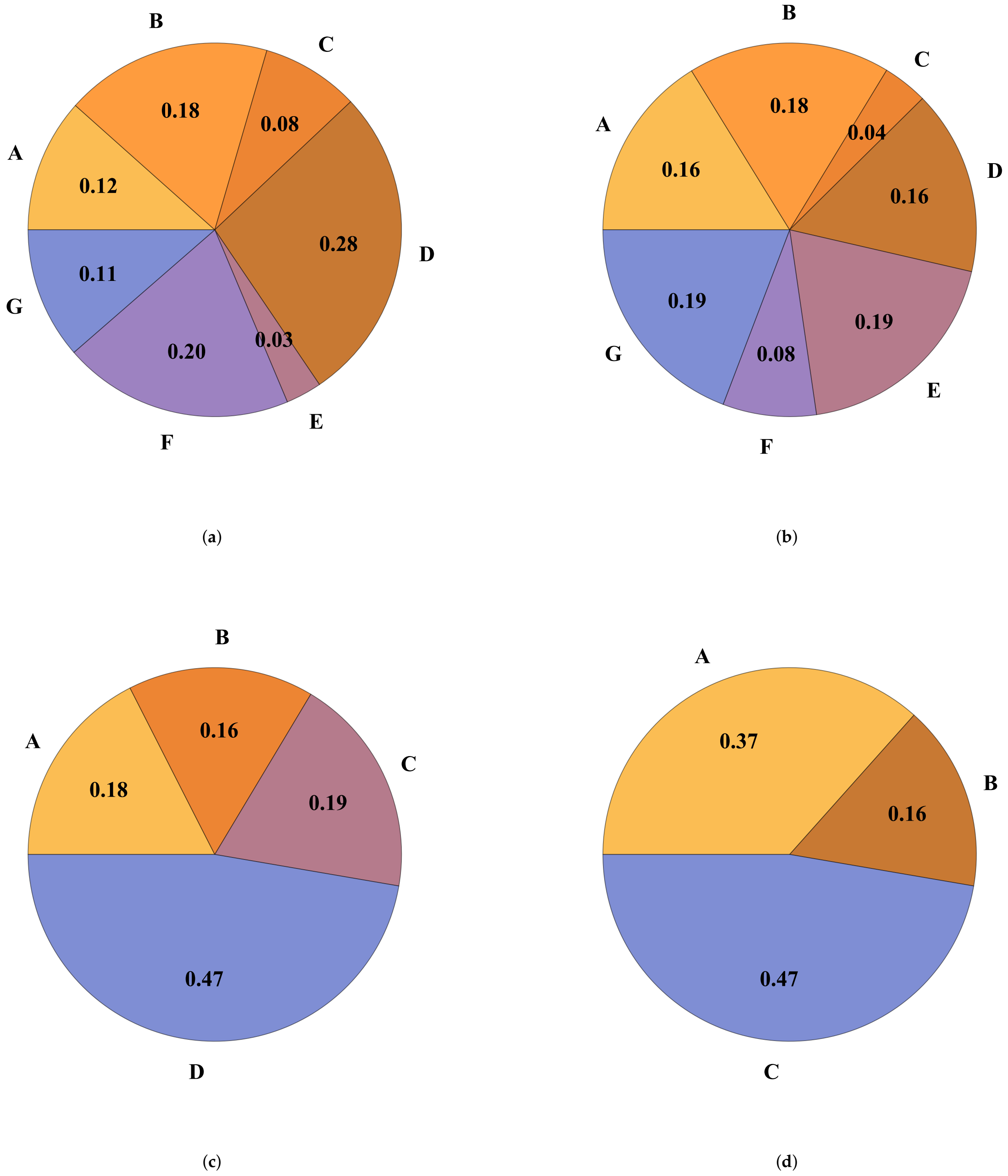

Figure S18. Pie charts representing the number of elements within each cluster, represented by the respective letters. (**a**) Experimental data clusters using NbC; (**b**–**d**) clusters of simulated data after parameter estimation using NbC clusters. (**b**) k-means; (**c**) NbC; (**d**) Gaussian Mixture.

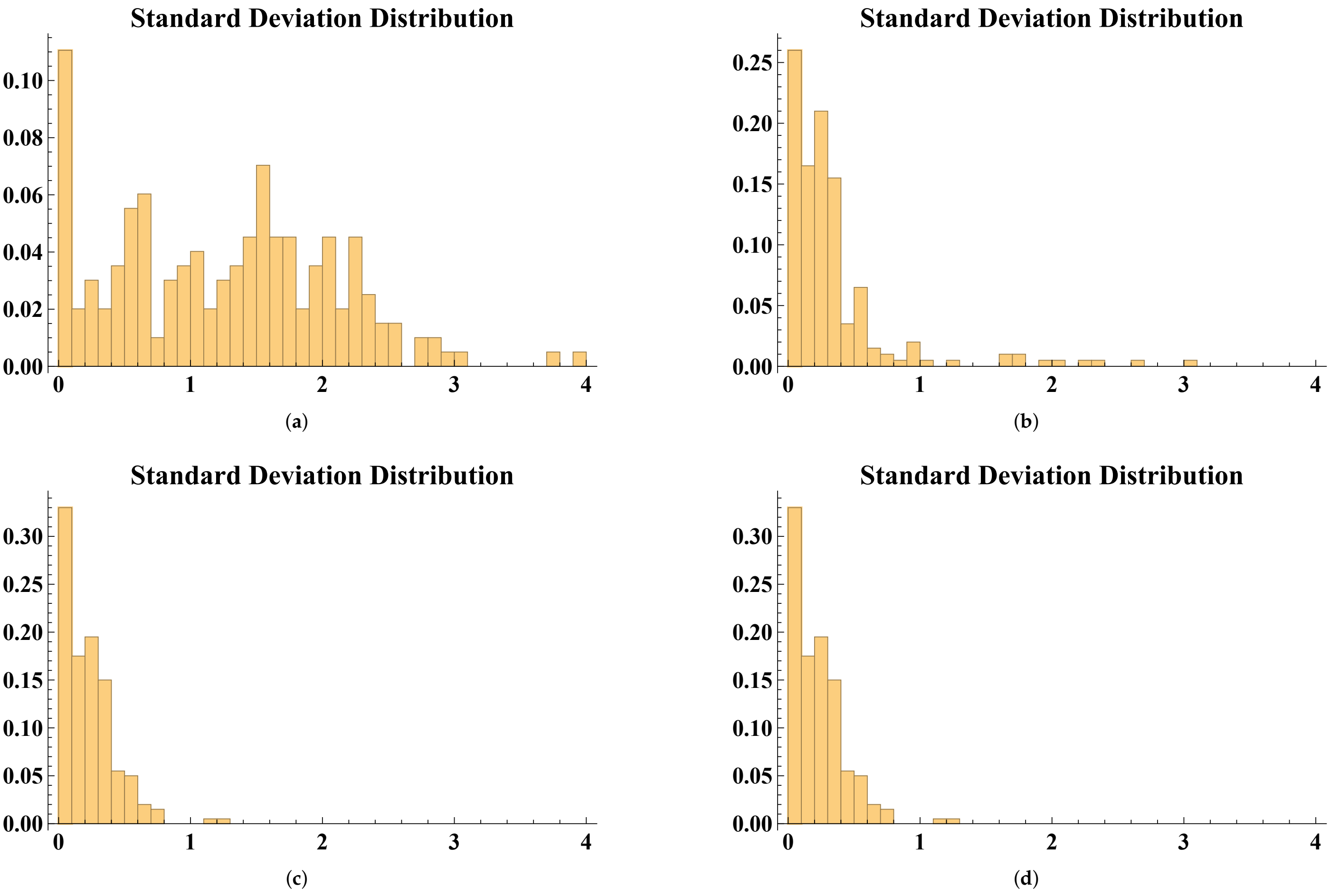

Figure S19. Standard deviation distribution of all genes within all clusters for each clustering method. (**a**) k-means applied to experimental data; (**b**–**d**) clustering of simulated data obtained after parameter estimation using k-means clusters. (**b**) k-means; (**c**) NbC; (**d**) Gaussian Mixture. The horizontal axis shows the standard deviation values, while the vertical axis represents normalized counts or frequencies to indicate probabilities.

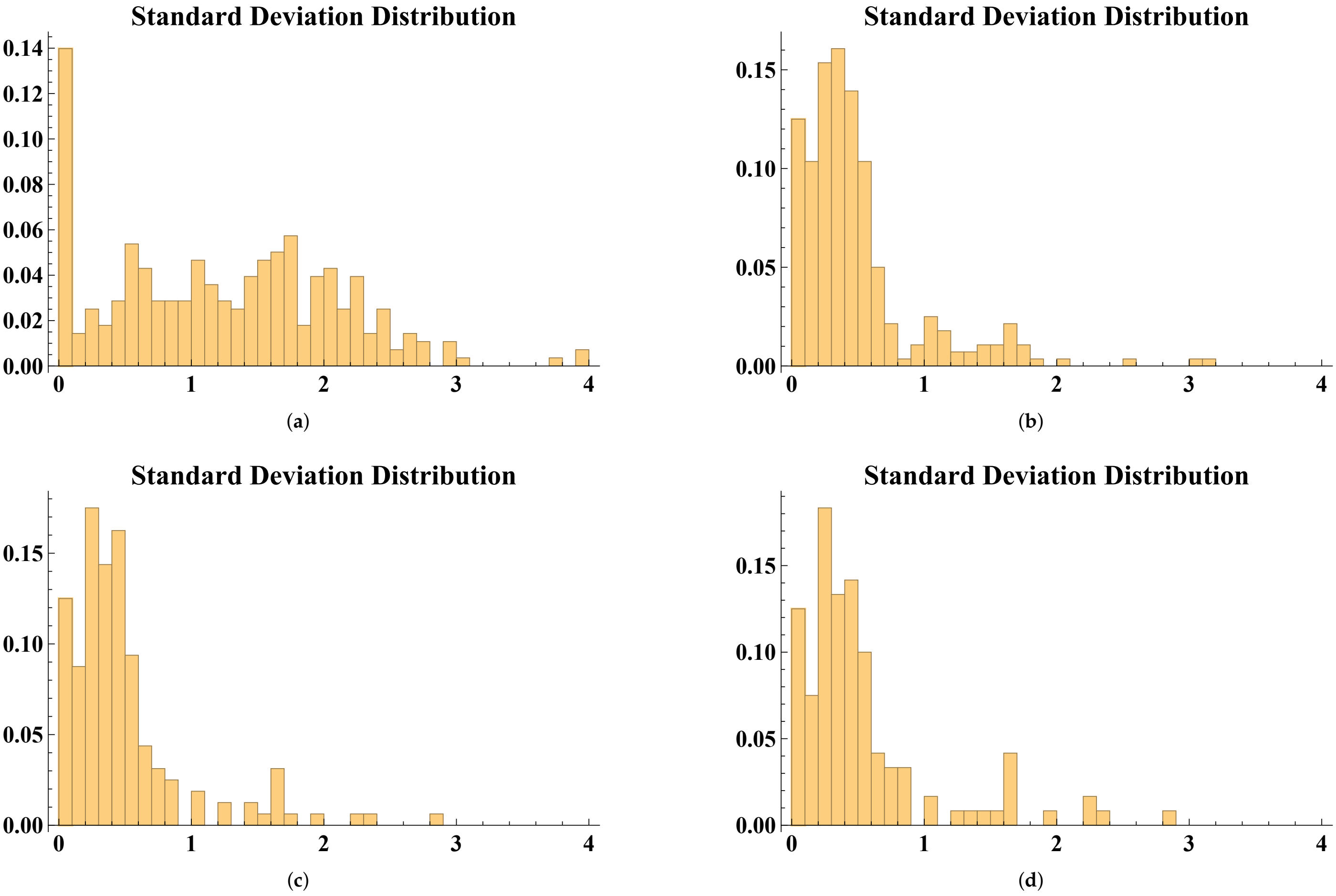

Figure S20. Standard deviation distribution of all genes within all clusters for each clustering method. (**a**) NbC applied to experimental data; (**b**–**d**) clustering of simulated data obtained after parameter estimation using NbC clusters. (**b**) k-means; (**c**) NbC; (**d**) Gaussian Mixture. The horizontal axis shows the standard deviation values, while the vertical axis represents normalized counts or frequencies to indicate probabilities.

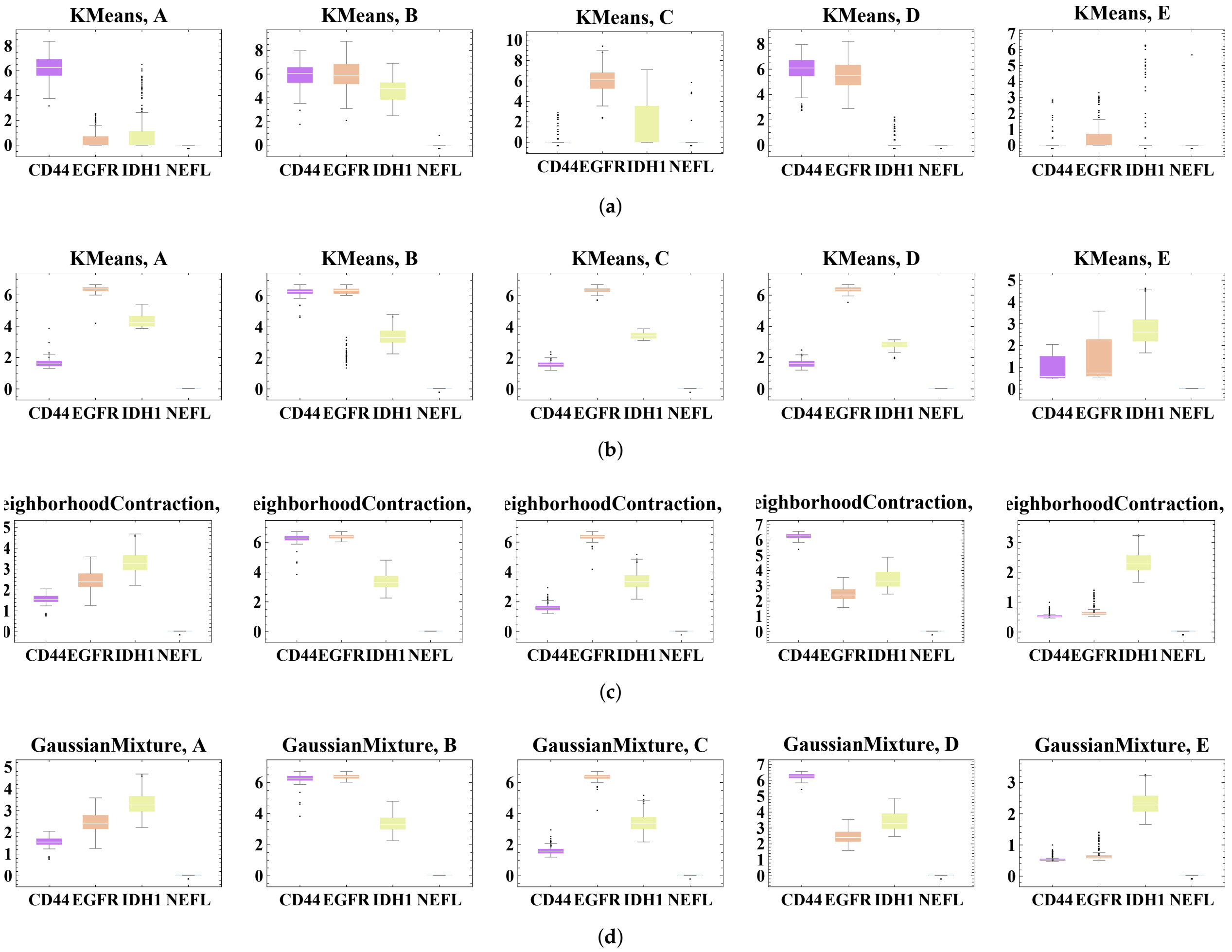

Figure S21. Boxplots of gene expression distributions for different clustering methods. (**a**) k-means applied to experimental data; (**b**) k-means applied to simulated data after parameter estimation using k-means clusters; (**c**) NbC applied to simulated data after parameter estimation using k-means clusters; (**d**) Gaussian Mixture applied to simulated data after parameter estimation using k-means clusters. The horizontal axis displays each considered marker gene, while the vertical axis represents the respective expression values.

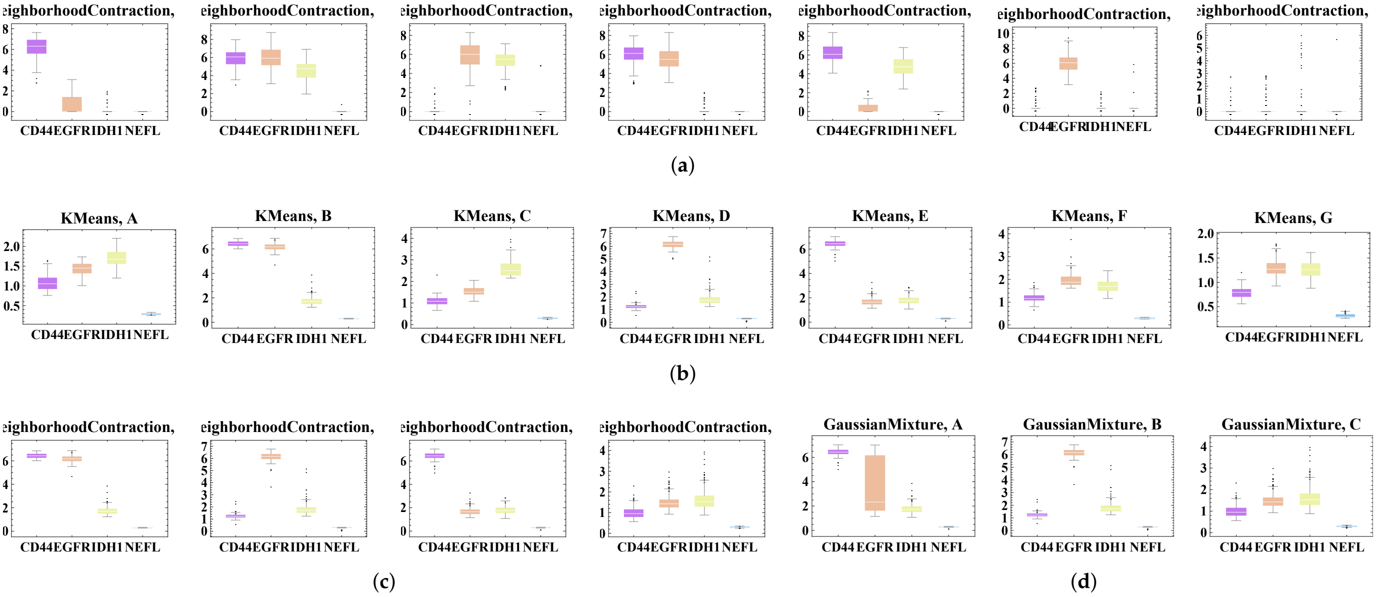

Figure S22. Boxplots of gene expression distributions for different clustering methods. (**a**) NbC applied to experimental data; (**b**) k-means applied to simulated data after parameter estimation using NbC clusters; (**c**) NbC applied to simulated data after parameter estimation using NbC clusters; (**d**) Gaussian Mixture applied to simulated data after parameter estimation using NbC clusters. The horizontal axis displays each considered marker gene, while the vertical axis represents the respective expression values.

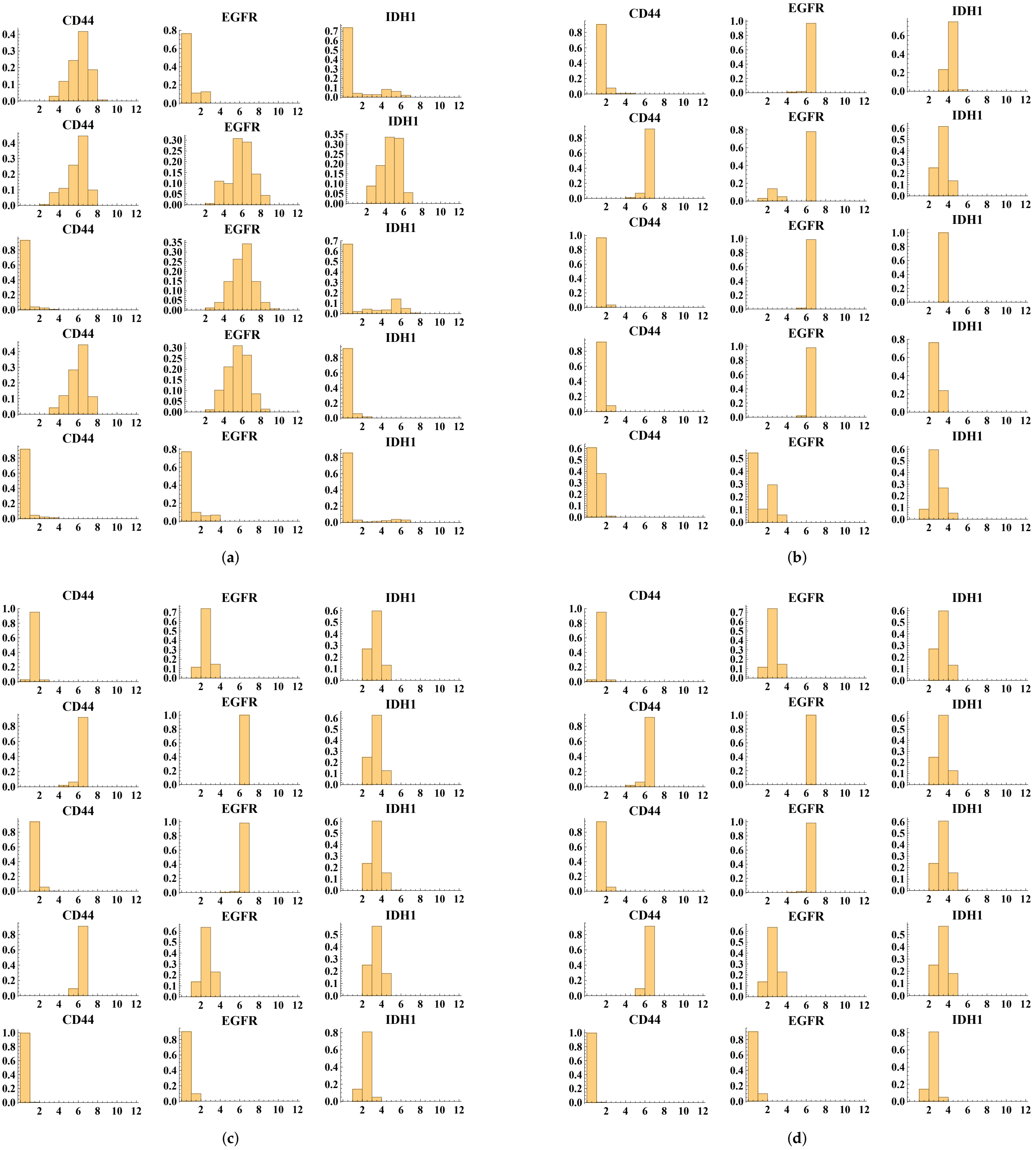

Figure S23. Gene expression distribution of markers for each cluster in different clustering methods. (**a**) k-means applied to experimental data; (**b**) k-means applied to simulated data after parameter estimation using k-means clusters; (**c**) NbC applied to simulated data after parameter estimation using k-means clusters; (**d**) Gaussian Mixture applied to simulated data after parameter estimation using k-means clusters. Each horizontal line shows the marker distribution for each respective cluster. For each case, the cluster identification ranges from A to E. The horizontal axis shows the expression values, while the vertical axis represents normalized counts or frequencies to indicate probabilities.

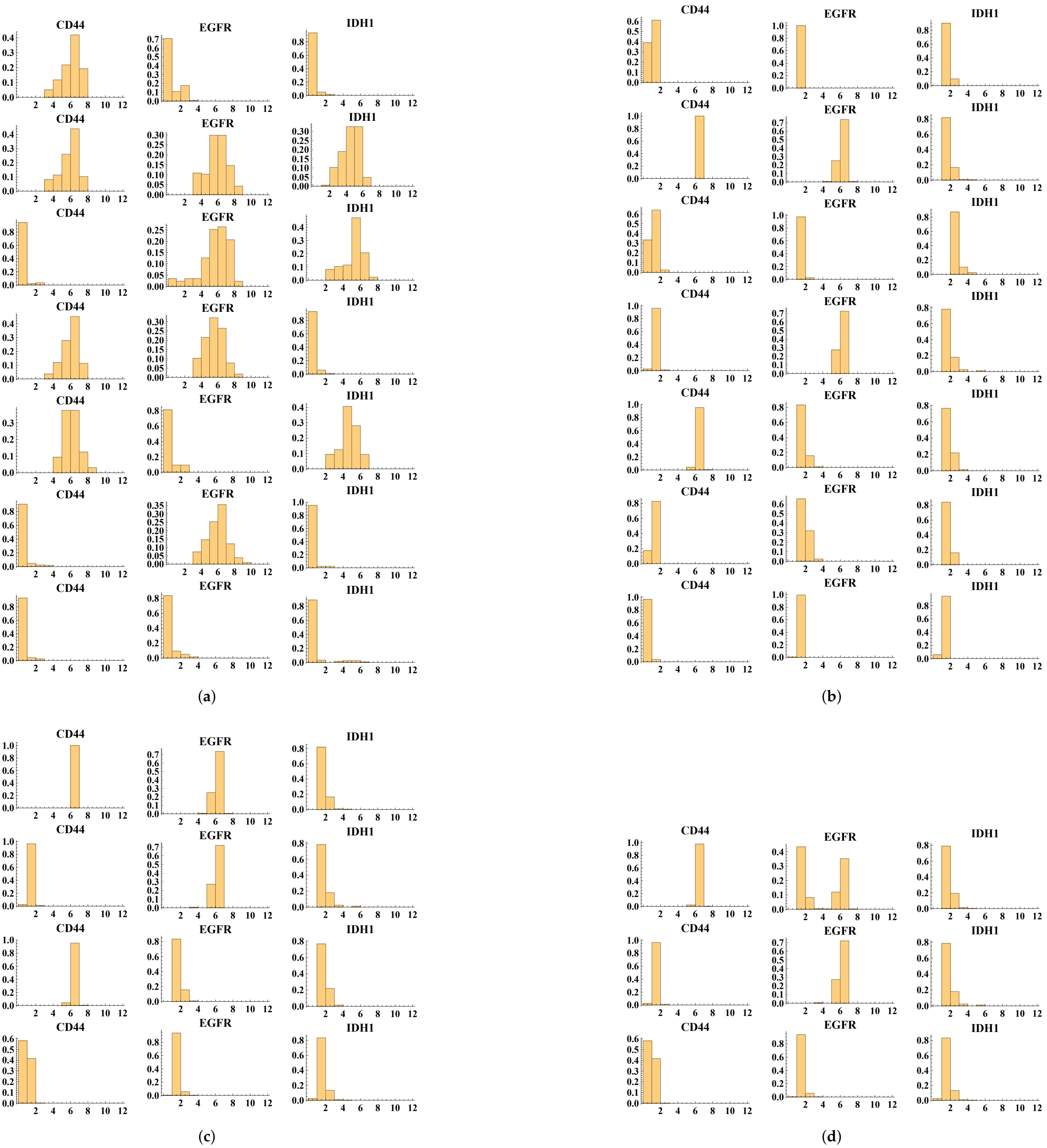

Figure S24. Gene expression distribution of markers for each cluster in different clustering methods. (**a**) NbC applied to experimental data; (**b**) k-means applied to simulated data after parameter estimation using NbC clusters; (**c**) NbC applied to simulated data after parameter estimation using NbC clusters; (**d**) Gaussian Mixture applied to simulated data after parameter estimation using NbC clusters. Each horizontal line shows the marker distribution for each respective cluster. For each case, the identification of the cluster ranges from A to its respective letter. The horizontal axis shows the expression values, while the vertical axis represents normalized counts or frequencies to indicate probabilities.

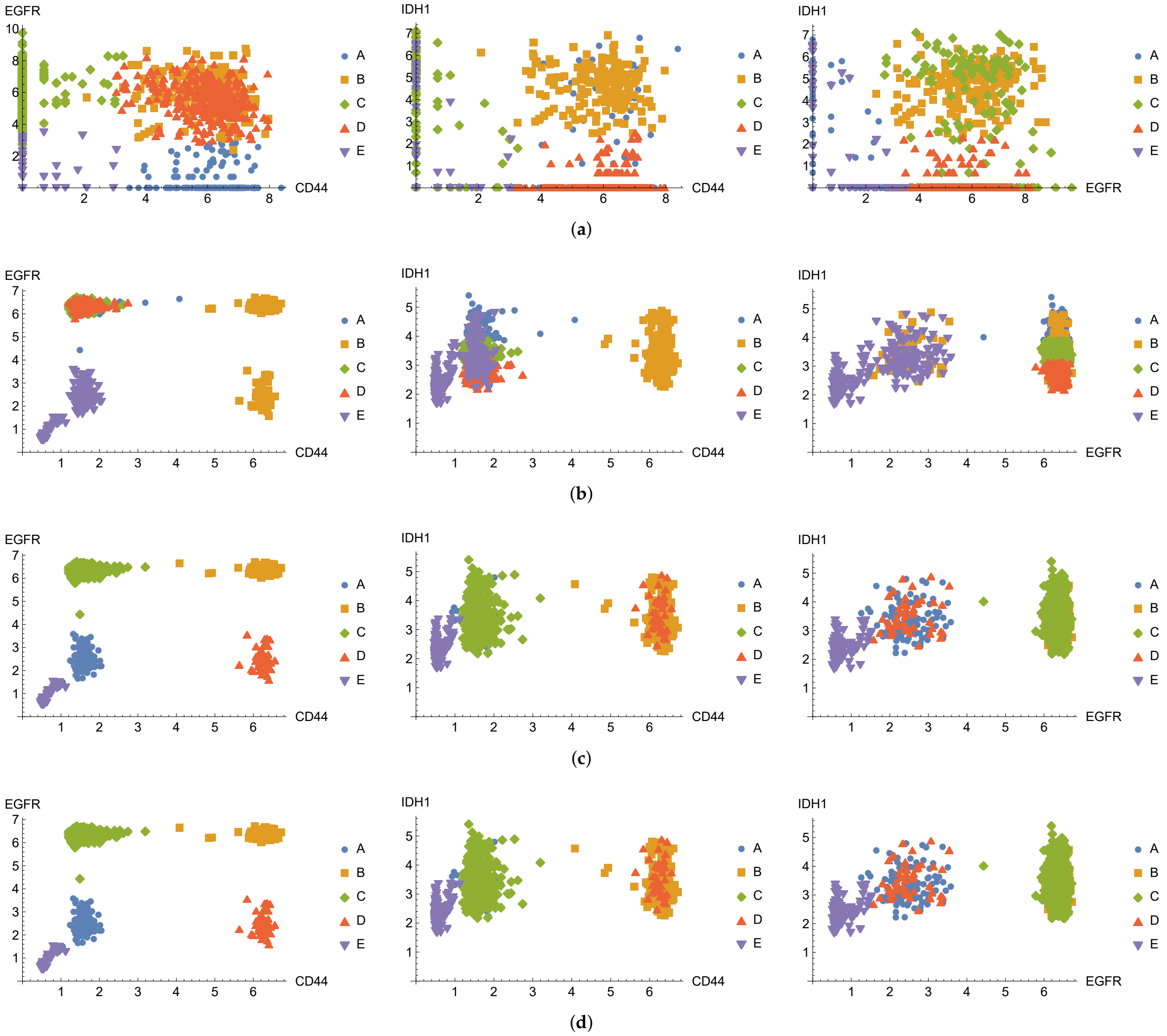

Figure S25. Scatter plots for combinations of marker gene expressions, excluding NEFL due to predominantly zero values. (**a**) Experimental data with five k-means clusters; (**b**–**d**) simulated data after parameter estimation using k-means clusters. (**b**) Clustering with k-means; (**c**) clustering with NbC; (**d**) clustering with Gaussian Mixture. Each scatter plot represents the relationship between the expressions of different marker genes in each clustering method. The horizontal and the vertical axes show the expression values of each marker gene, while the different colors and shapes represent their corresponding cluster.

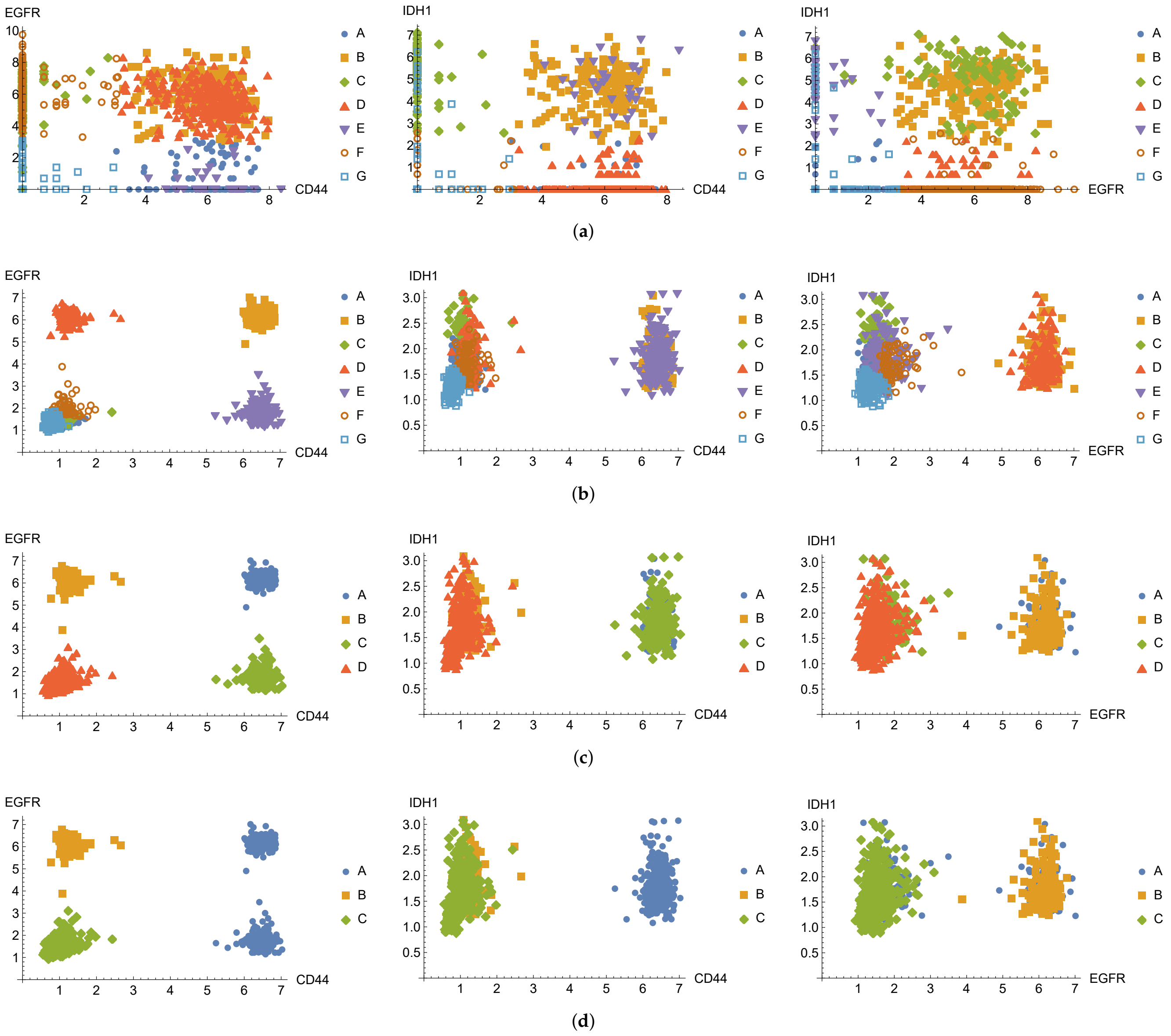

Figure S26. Scatter plots for combinations of marker gene expressions, excluding NEFL due to predominantly zero values. (**a**) Experimental data with seven NbC clusters; (**b**–**d**) simulated data after parameter estimation using NbC clusters. (**b**) Clustering with k-means; (**c**) clustering with NbC; (**d**) clustering with Gaussian Mixture. Each scatter plot represents the relationship between the expressions of different marker genes in each clustering method. The horizontal and the vertical axes show the expression values of each marker gene, while the different colors and shapes represent their corresponding cluster.

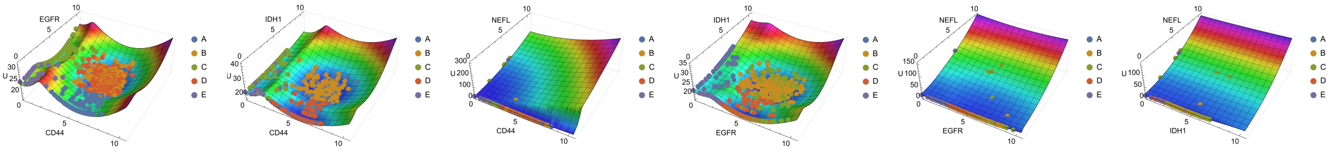

(**a**)

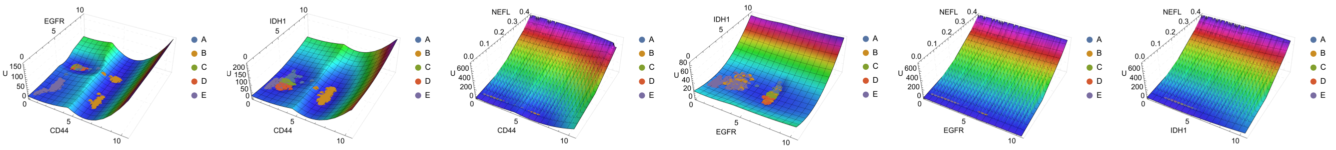

(**b**)

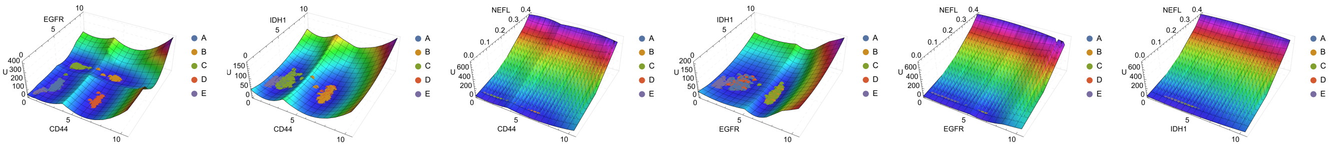

(**c**)

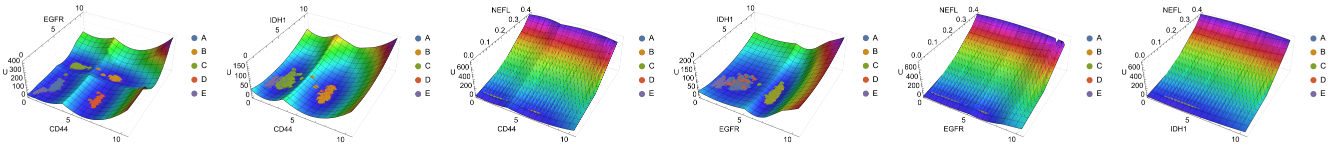

(**d**)

Figure S27. (Epi)genetic landscapes for experimental and simulated data, with experimental and simulated points overlaid for compatibility visualization. The colors of the landscape represent the vertical axis values, highlighting the representation of the 3D dimension. (**a**) Landscape for experimental data with k-means clusters; (**b**) landscape for simulated data after parameter estimation and clustering with k-means; (**c**) landscape for simulated data after parameter estimation and clustering with NbC; (**d**) landscape for simulated data after parameter estimation and clustering with Gaussian Mixture. These landscapes visually represent the compatibility between experimental and simulated data in each clustering method. The horizontal axes show the expression values of each marker gene, while the vertical axis represents the values of the landscape. The different colors and shapes correspond to the respective clusters.

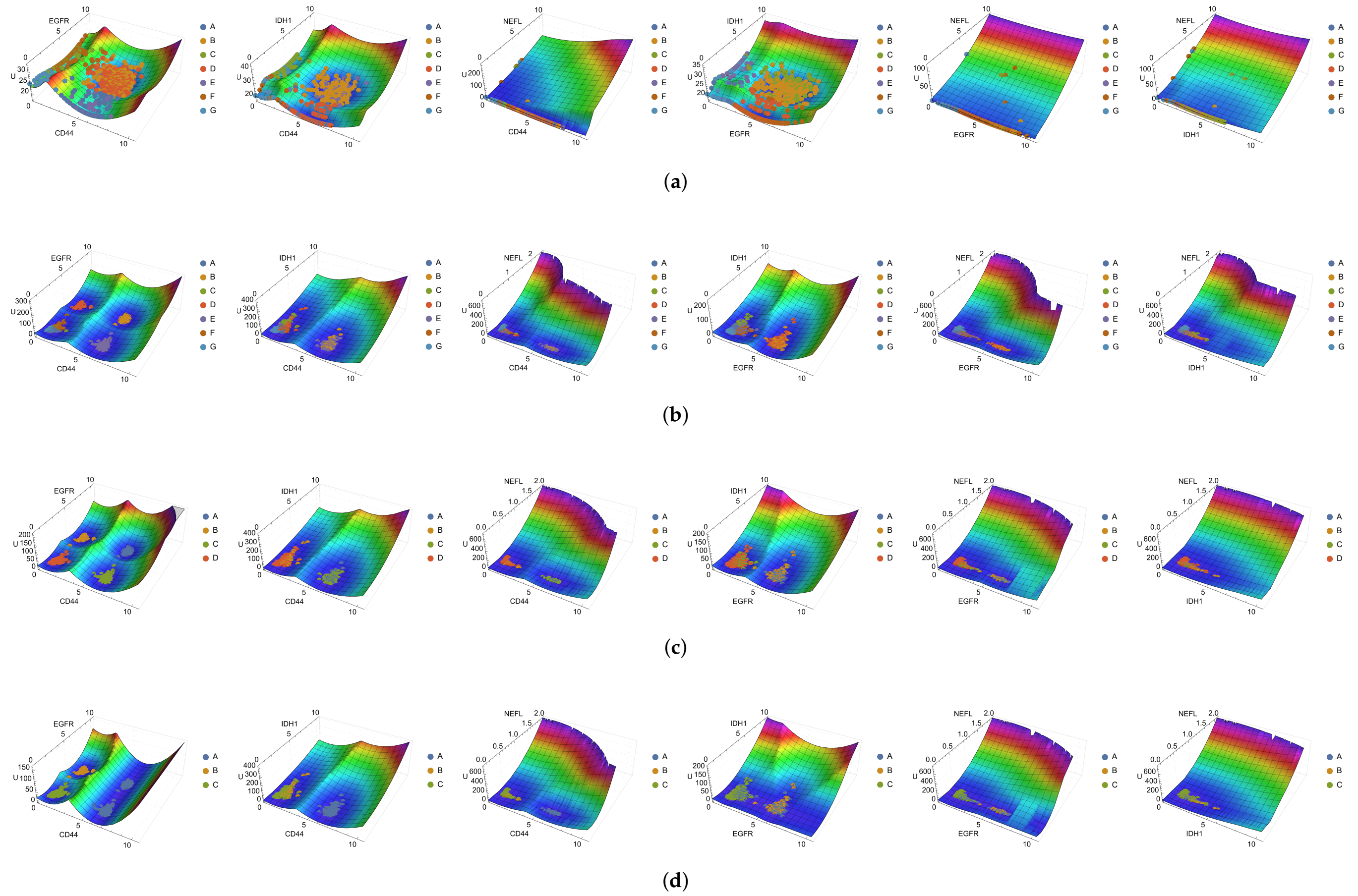

Figure S28. (Epi)genetic landscapes for experimental and simulated data, with experimental and simulated points overlaid for compatibility visualization. (**a**) Landscape for experimental data with NbC clusters; (**b**) landscape for simulated data after parameter estimation and clustering with k-means; (**c**) landscape for simulated data after parameter estimation and clustering with NbC; (**d**) landscape for simulated data after parameter estimation and clustering with Gaussian Mixture. These landscapes visually represent the compatibility between experimental and simulated data in each clustering method. The horizontal axes show the expression values of each marker gene, while the vertical axis represents the values of the landscape. The different colors and shapes correspond to the respective clusters.

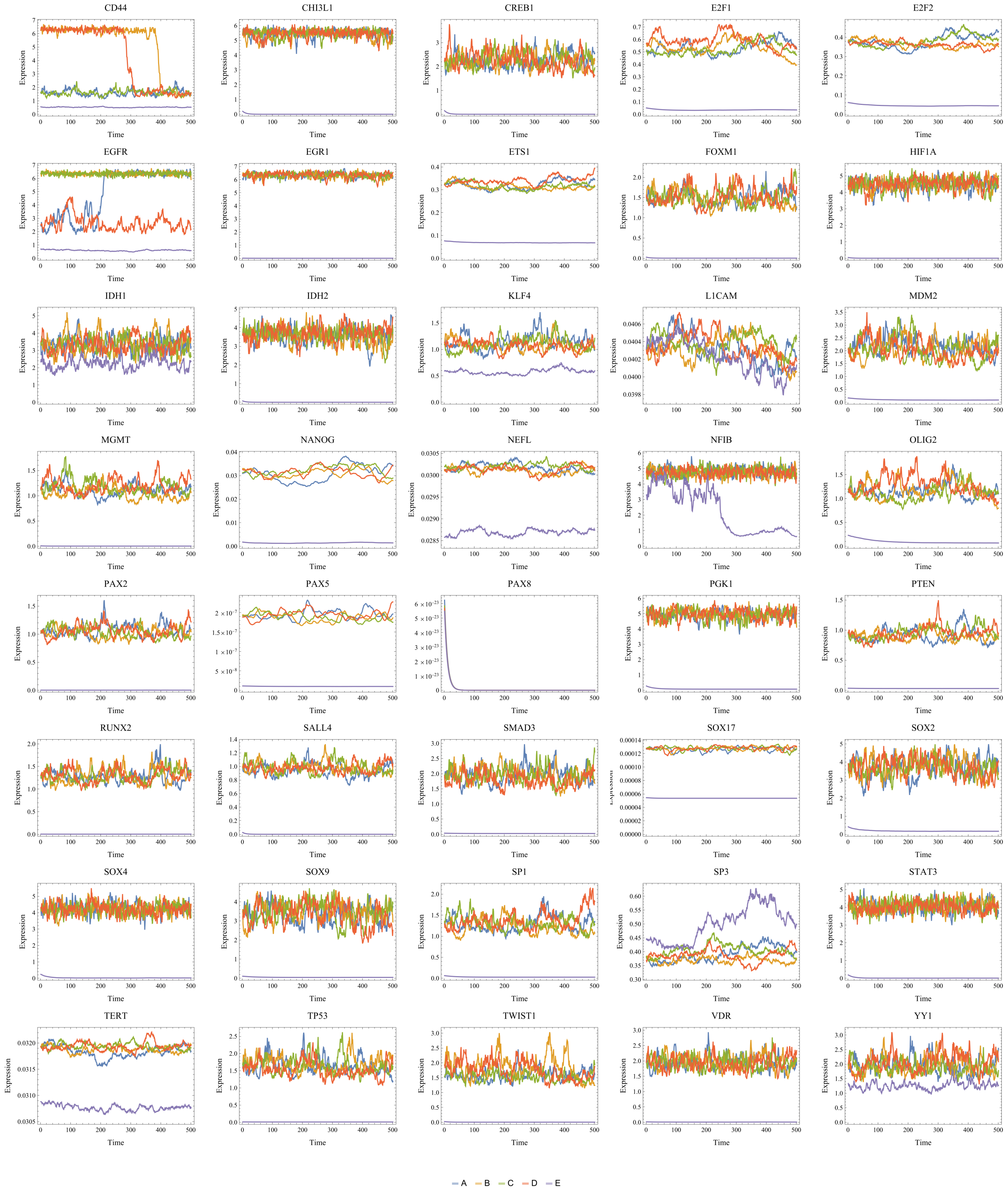

Figure S29. Trajectory plots for each basin, showcasing the system's dynamics. Each panel represents a different gene, with five trajectories, one for each cluster. The horizontal axis represents time, and the vertical axis shows the expression level of the gene. The trajectories illustrate the time evolution from initial conditions as the centroid of each cluster. These visualizations provide insights into the internal dynamics of each basin and help identify potential transitions between clusters.

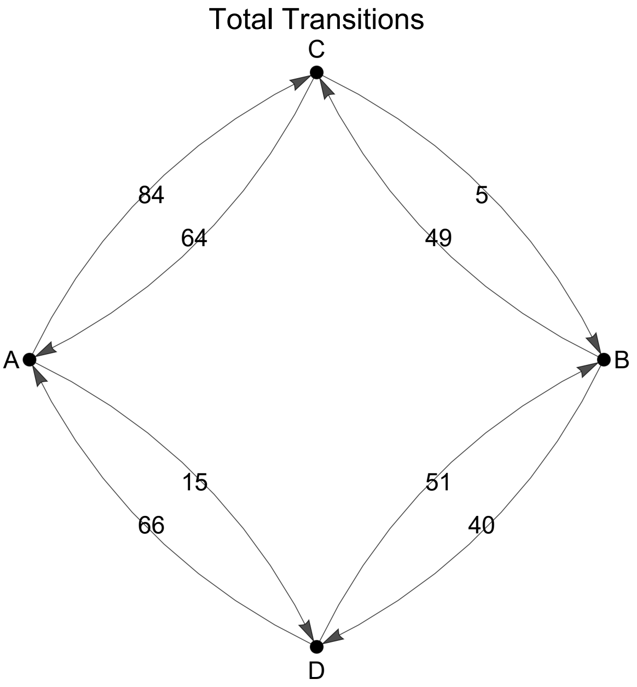

(**a**) (**b**)

Figure S30. Transition graphs illustrating the connections between different basins, represented by each cluster letter (A to D). (**a**) Graph representation of transitions between basins, with vertices representing basins and edges representing observed transitions. (**b**) The same transition graph as in (**a**), but with edge weights representing the probabilities of transitions between basins. These visualizations provide insights into the potential transition pathways and their relative likelihood within the system.

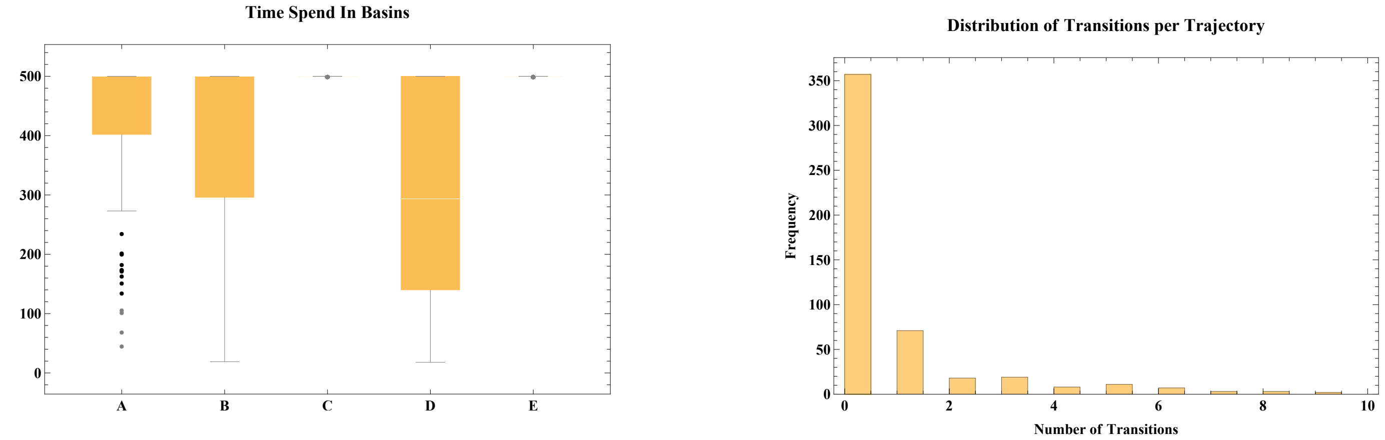

(**a**) (**b**)

Figure S31. Analysis of time spent in basins before a transition and the frequency of transitions per trajectory. (**a**) Box plots of the distribution of time spent in each basin across all trajectories before they present a transition, providing insights into the relative stability of different basins. The vertical axis represents the time spent in the basin, while the horizontal axis the correspondent basins. (**b**) Histogram showing the frequency of transitions between basins in each trajectory, highlighting that most trajectories do not present any transition, and those that do tend to have a small number of transitions. The vertical axis shows the frequency of each number of transitions per trajectory, while the horizontal axis the number of transitions per trajectory. These plots help to assess the inter-basin dynamics.

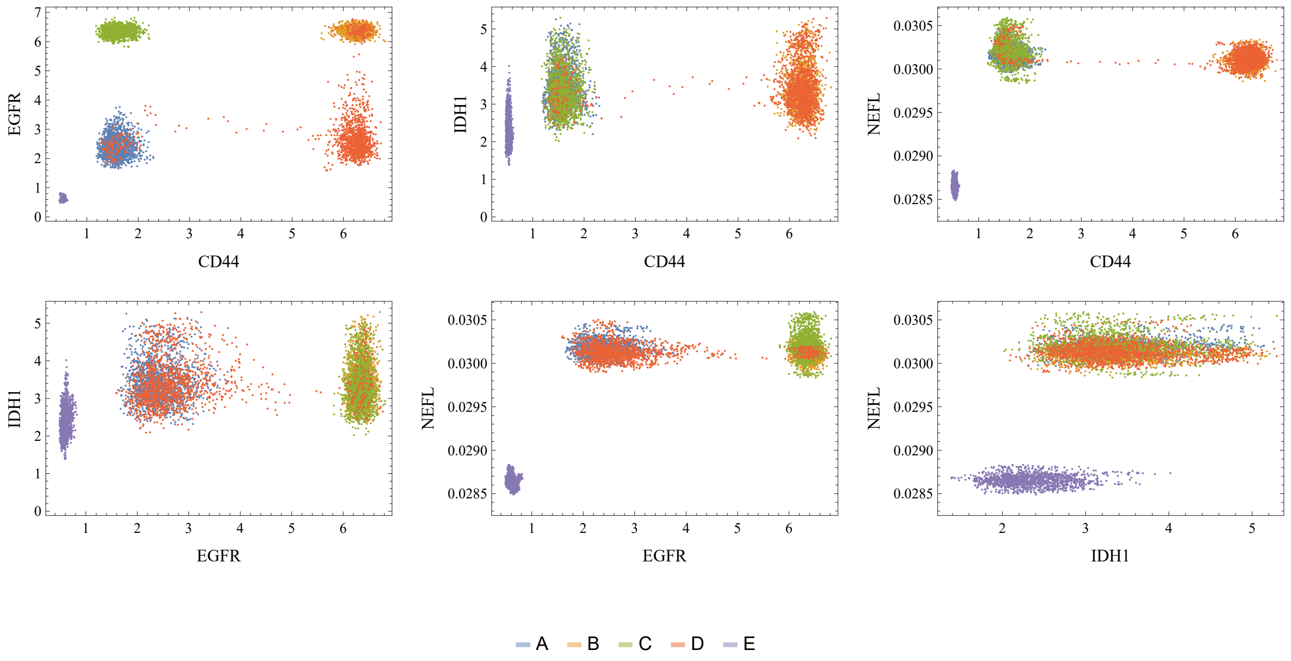

Figure S32. Two-dimensional visualization of the trajectories in the markers combination space. The horizontal and vertical axes display the expression values of each marker gene. Each point represents a single time step of three considered trajectories. Each color/letter indicates its respective basin. This figure helps to illustrate the trajectories' paths and the system's dynamics in a simplified 2D space.

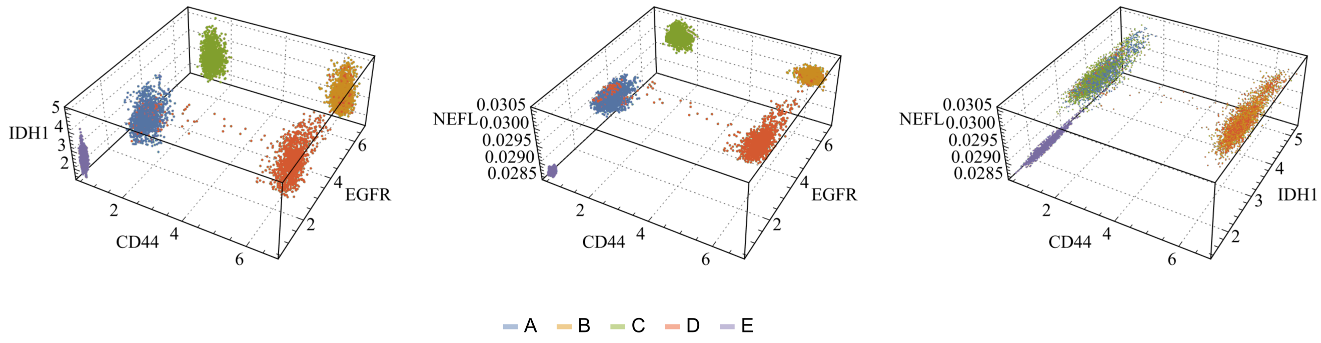

Figure S33. Three-dimensional visualization of the trajectories in the markers combination space. All axes display the expression values of each marker gene. Each point represents a single time step of three considered trajectories. Each color/letter indicates its respective basin. This figure offers a more detailed view of the system's dynamics and trajectories in a 3D space.

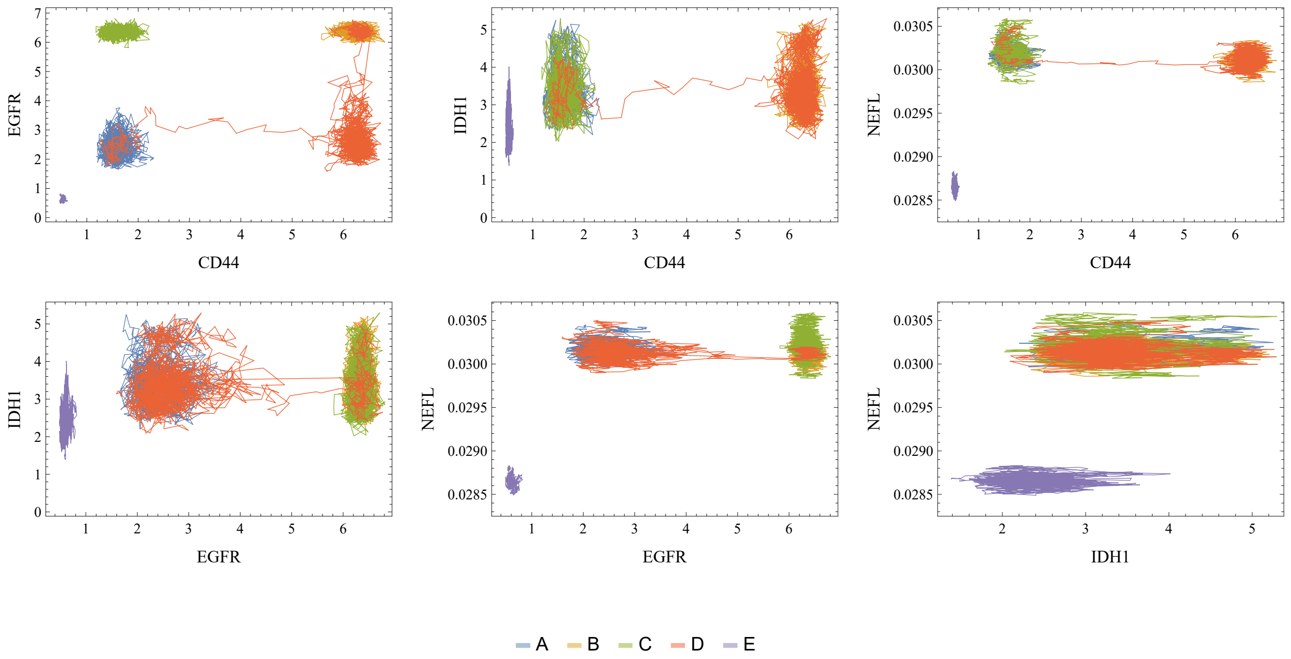

Figure S34. Two-dimensional visualization of full trajectories in the markers combination space. The horizontal and vertical axes display the expression values of each marker gene. Each line represents an entire time of the three considered trajectories. Each color/letter indicates its respective basin. This figure provides an overview of the paths and dynamics of the system's trajectories in a 2D space.

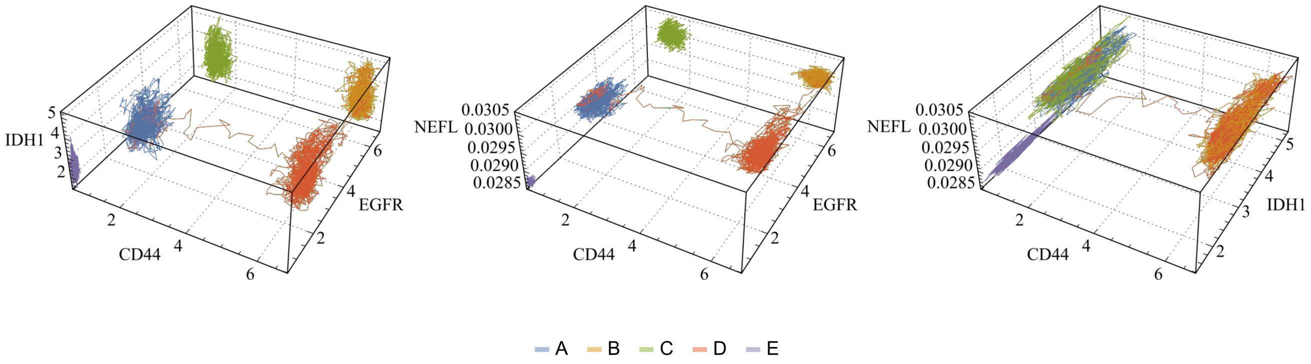

Figure S35. Three-dimensional visualization of full trajectories in the markers combination space. All axes display the expression values of each marker gene. Each line represents an entire time of the three considered trajectories. Each color/letter indicates its respective basin. This figure offers a comprehensive view of the system's dynamics and trajectories in a 3D space.

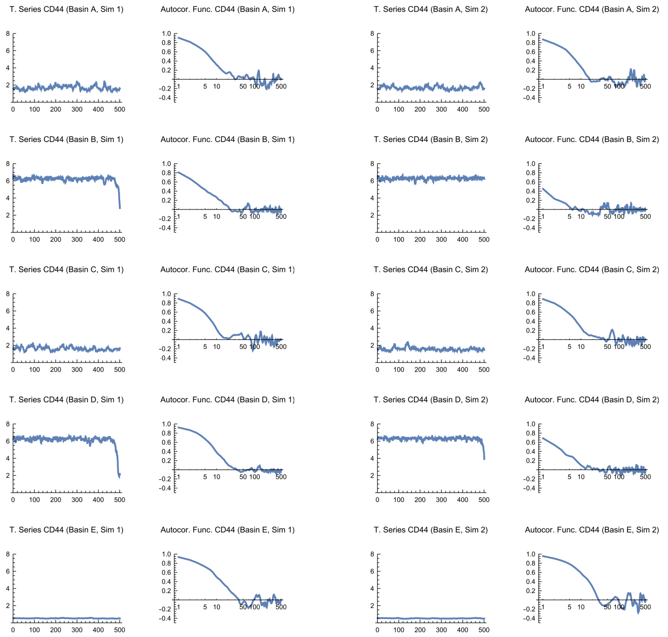

(**a**)

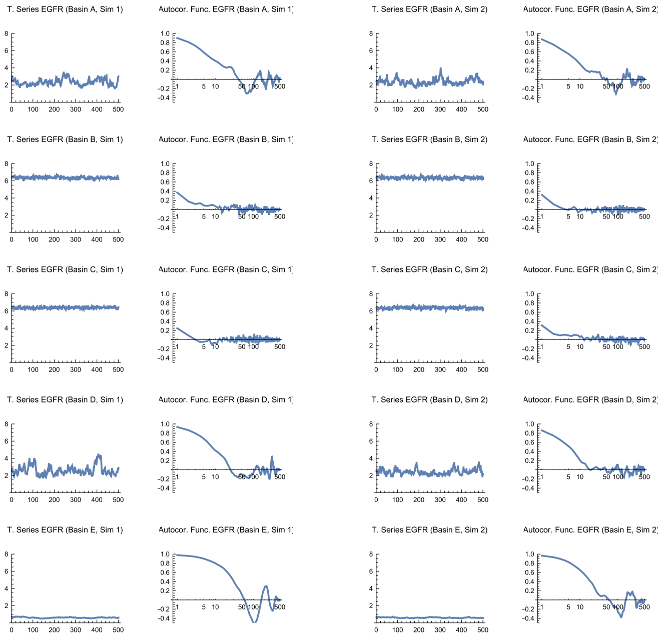

(**b**)

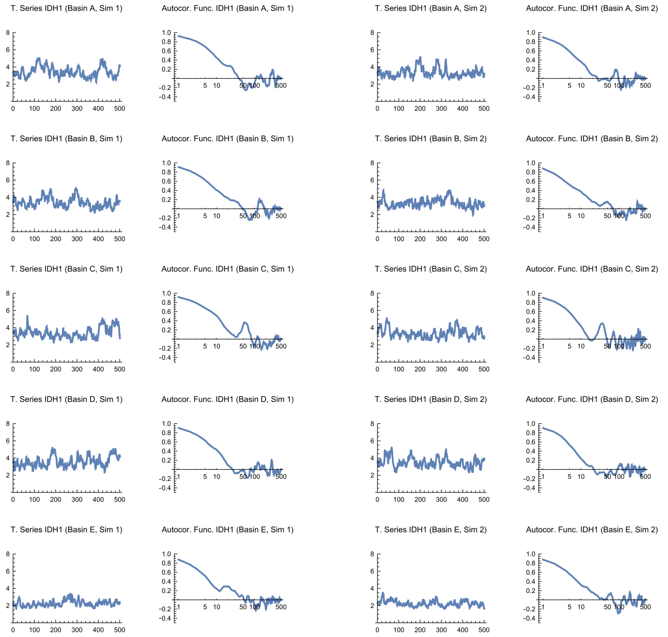

(**c**)

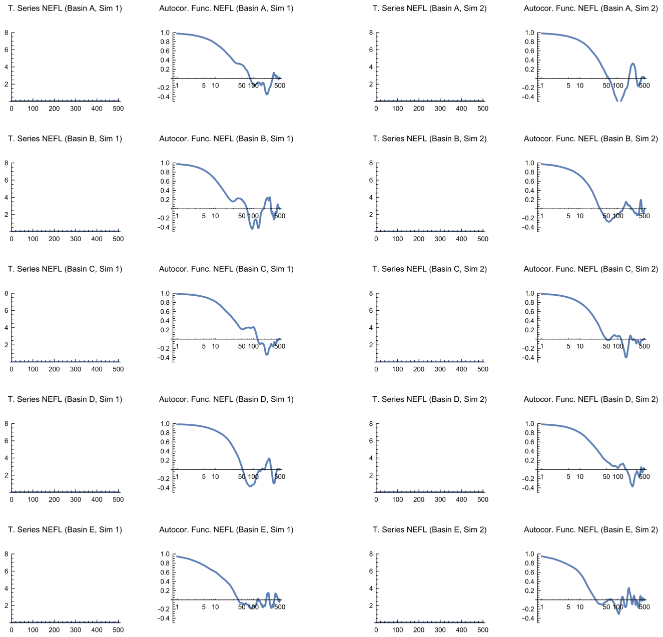

(**d**)

Figure S36. Autocorrelation analysis of time series data for different genes, basins, and repetitions. (**a**–**d**) Autocorrelation plots for four representative genes, illustrating the dependence structure of the time series data. Each pair of plots within (**a**–**d**) includes a time series plot (**left**) with the horizontal axis representing time and the vertical axis representing expression values and an autocorrelation plot (**right**) with the horizontal axis representing time lags and the vertical axis representing autocorrelation values. This figure helps to assess the temporal dependence of gene expressions and the potential impact of time series structure on the autocorrelation analysis.

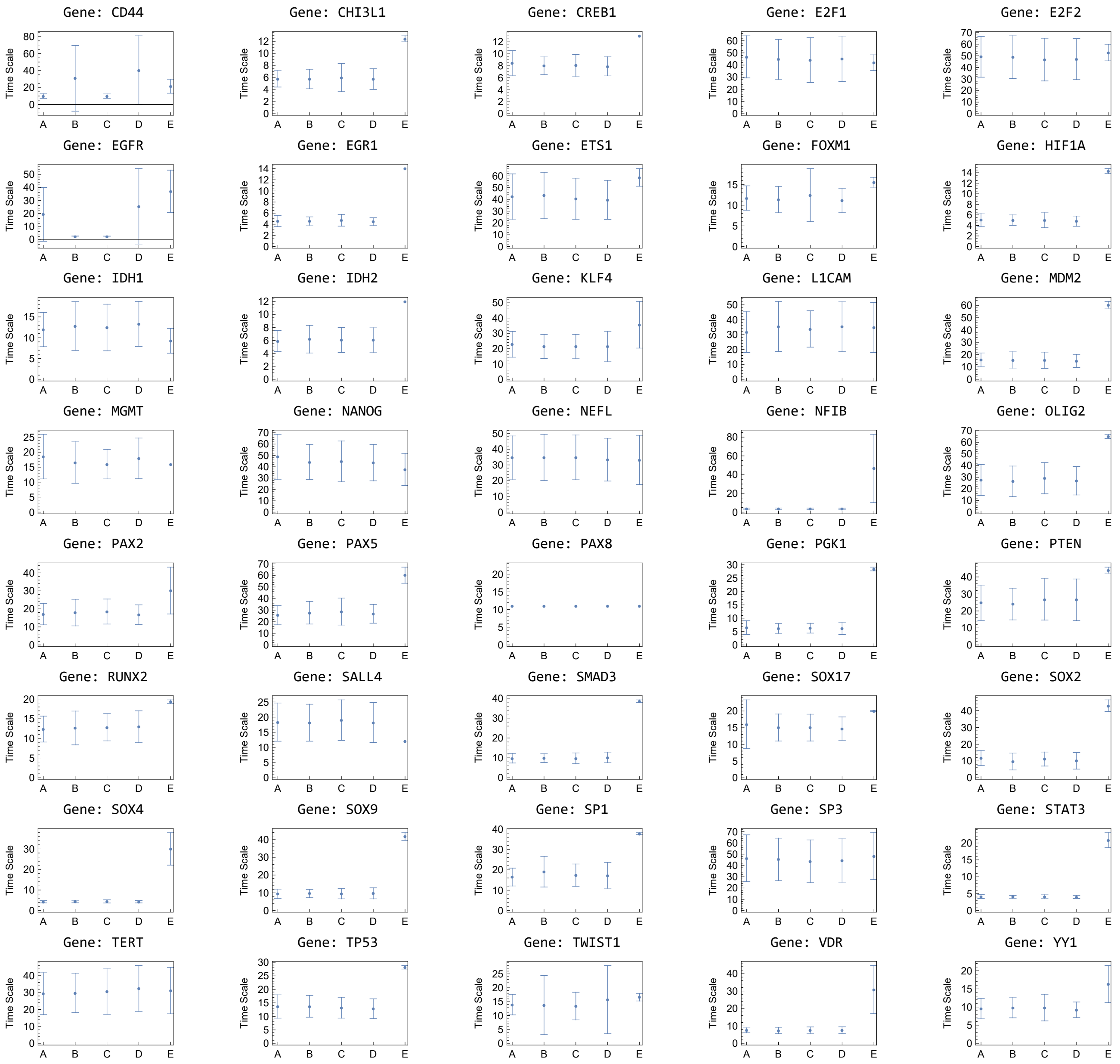

Figure S37. Distribution of timescales for different genes and their basins. The horizontal axis represents the cluster labels, and the vertical axis represents the timescale values, defined as the minimum time lag at which the autocorrelation falls below e^{-1}. The figure presents the distribution of timescales for each gene, with error bars indicating the standard deviation within all repetitions. This visualization helps to understand the characteristic timescales within and across different genes, providing insights into the internal dynamics and possible transition behaviors of the system.

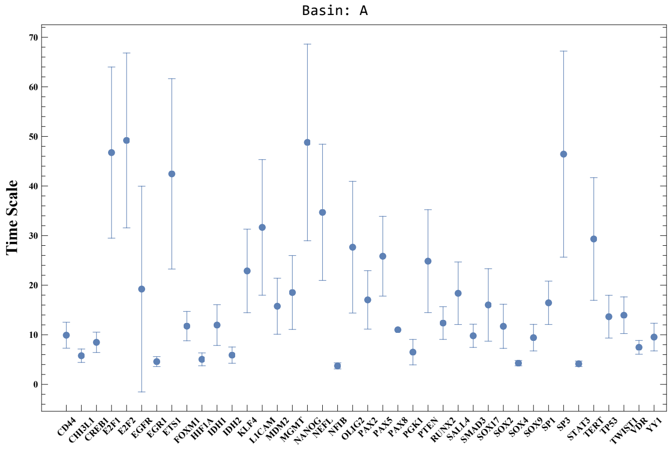

(**a**)

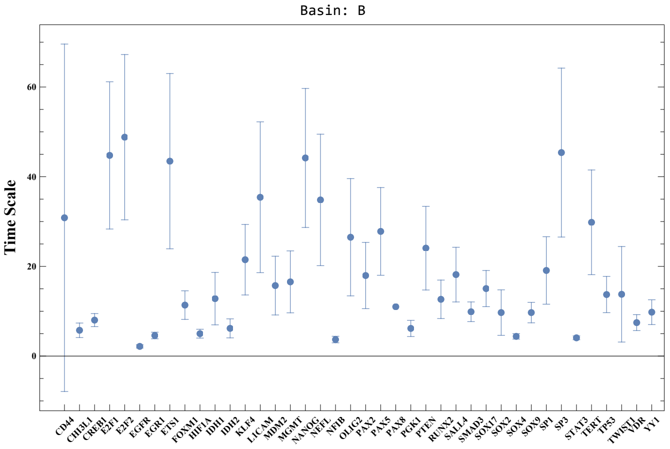

(**b**)

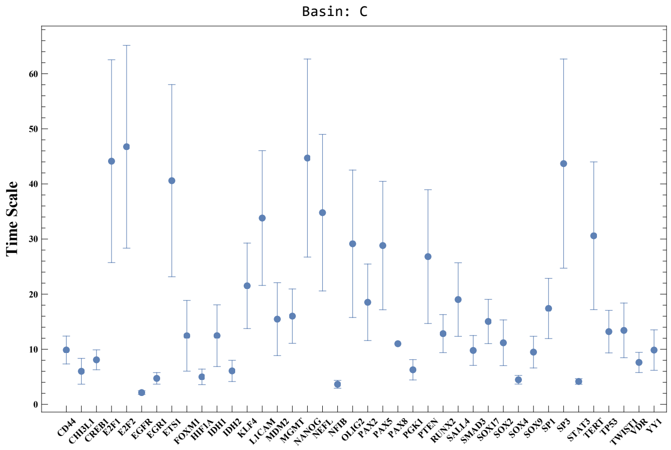

(**c**)

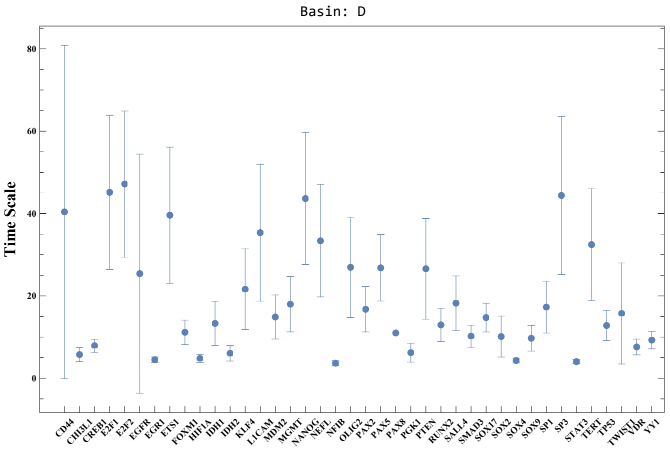

(**d**)

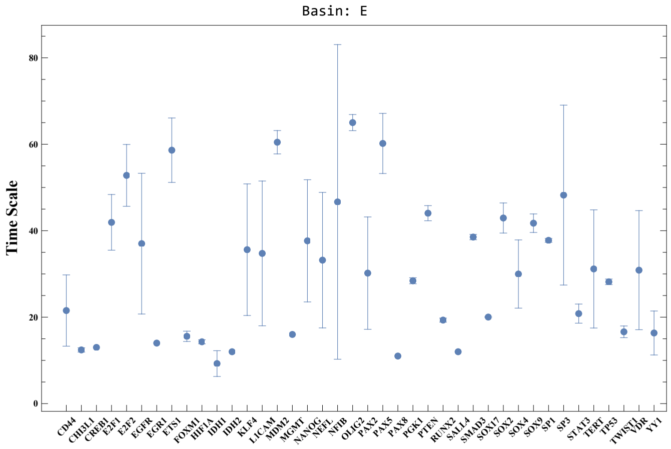

(**e**)

Figure S38. Timescale distributions for different genes within their basins (subfigures a to e). The horizontal axis represents the gene labels, and the vertical axis represents the timescale values, defined as the minimum time lag at which the autocorrelation falls below e^{-1}. The figure emphasizes the differences within each basin, showcasing the distinct characteristics of each gene's timescale distribution across different basins.

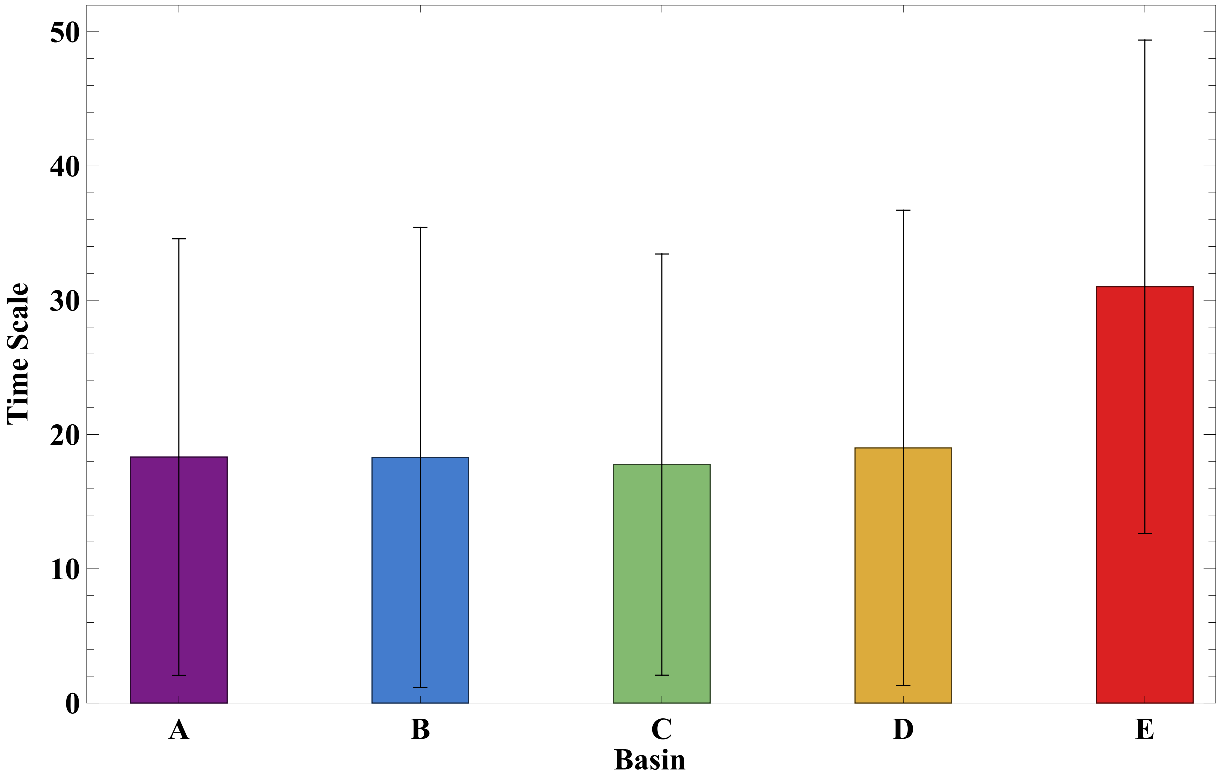

Figure S39. Final average timescales of each basin across all genes and repetitions. The horizontal axis represents the cluster labels (A to E), and the vertical axis represents each basin's final average timescale values.

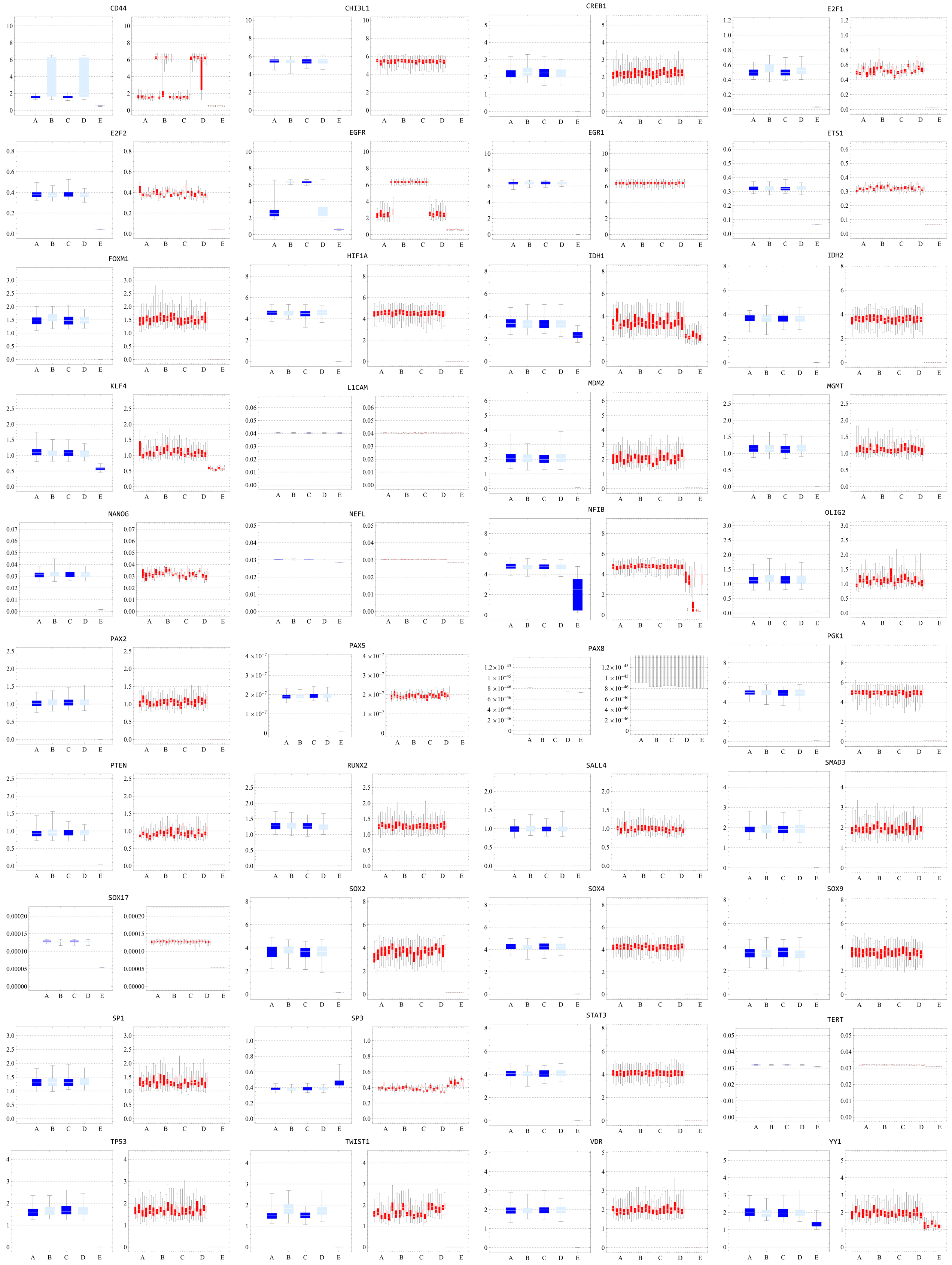

Figure S40. Comparison of time and sample averages for various clusters and genes using boxplots. The horizontal axis represents the cluster labels, and the vertical axis shows the expression values. The figure displays two sets of plots: the left set shows the sample average of 100 samples at the final time interval, while the right set represents the time average considering 10 trajectories from time 30

to 50 (steps 300 to 500). The boxplots illustrate the distribution of expression values within each cluster, with the box representing the interquartile range (IQR), the line inside the box showing the median, and the whiskers extending to the minimum and maximum data points within 1.5 times the IQR. This comparison helps to assess the compatibility between time and sample averages, supporting our hypothesis and providing insights into potential discrepancies between different basins and genes.
